# Myocardial Involvement After Hospitalization for COVID-19 Complicated by Troponin Elevation: A Prospective, Multicenter, Observational Study

**DOI:** 10.1161/CIRCULATIONAHA.122.060632

**Published:** 2023-01-31

**Authors:** Jessica Artico, Hunain Shiwani, James C. Moon, Miroslawa Gorecka, Gerry P. McCann, Giles Roditi, Andrew Morrow, Kenneth Mangion, Elena Lukaschuk, Mayooran Shanmuganathan, Christopher A. Miller, Amedeo Chiribiri, Sanjay K. Prasad, Robert D. Adam, Trisha Singh, Chiara Bucciarelli-Ducci, Dana Dawson, Daniel Knight, Marianna Fontana, Charlotte Manisty, Thomas A. Treibel, Eylem Levelt, Ranjit Arnold, Peter W. Macfarlane, Robin Young, Alex McConnachie, Stefan Neubauer, Stefan K. Piechnik, Rhodri H. Davies, Vanessa M. Ferreira, Marc R. Dweck, Colin Berry, OxAMI (Oxford Acute Myocardial Infarction Study) Investigators; COVID-HEART Investigators, John P. Greenwood

**Affiliations:** 1 Institute of Cardiovascular Science (J.A., H.S., J.C.M., R.D.A., C.M., T.A.T., R.H.D.), University College London, UK.; 2 Division of Medicine, Royal Free Hospital (D.K., M.F.), University College London, UK.; 3 Institute of Cardiovascular and Metabolic Medicine, University of Leeds, and Leeds Teaching Hospitals NHS Trust, UK (M.G., E. Levelt, J.P.G.).; 4 University of Leicester and the National Institute for Health and Care Research (NIHR) Leicester Biomedical Research Centre, Glenfield Hospital, UK (G.P.M., R.A.).; 5 Institute of Cardiovascular and Medical Sciences and British Heart Foundation Glasgow Cardiovascular Research Centre (G.R., A. Morrow, K.M., C.B.), Institute of Health and Wellbeing, University of Glasgow, UK.; 6 Electrocardiology Core Laboratory (P.W.M.), Institute of Health and Wellbeing, University of Glasgow, UK.; 7 Robertson Centre for Biostatistics (R.Y., A. McConnachie), Institute of Health and Wellbeing, University of Glasgow, UK.; 8 Division of Cardiovascular Medicine, Radcliffe Department of Medicine, Oxford Centre for Clinical Magnetic Resonance Research, British Heart Foundation Centre of Research Excellence, Oxford NIHR Biomedical Research Centre, University of Oxford, UK (E. Lukaschuk, M.S., S.N., S.K.P., V.M.F.).; 9 Division of Cardiovascular Sciences, School of Medical Sciences, Faculty of Biology, Medicine and Health, University of Manchester, UK (C.A.M.).; 10 School of Biomedical Engineering and Imaging Sciences, King’s College London, BHF Centre of Excellence and the NIHR Biomedical Research Centre at Guy’s and St Thomas’ NHS Foundation Trust, The Rayne Institute, St Thomas’ Hospital, London, UK (A.C., C.B.-D.).; 11 National Heart and Lung Institute, Imperial College, London, UK (S.K.P.).; 12 University of Edinburgh and British Heart Foundation Centre for Cardiovascular Science, UK (T.S., M.R.D.).; 13 Royal Brompton and Harefield Hospitals and Guys’ and St Thomas NHS Trust, London, UK (C.B.-D.).; 14 Bristol Heart Institute, University Hospitals Bristol and Weston NHS Trust, Bristol, UK (C.B.-D.).; 15 Department of Cardiology, Aberdeen Cardiovascular and Diabetes Centre, Aberdeen Royal Infirmary and University of Aberdeen, UK (D.D.).

**Keywords:** cardiovascular diseases, coronavirus, COVID-19, magnetic resonance imaging, myocardial infarction, troponin

## Abstract

Acute myocardial injury in hospitalized patients with coronavirus disease 2019 (COVID-19) has a poor prognosis. Its associations and pathogenesis are unclear. Our aim was to assess the presence, nature, and extent of myocardial damage in hospitalized patients with troponin elevation.

**Methods:**

Across 25 hospitals in the United Kingdom, 342 patients with COVID-19 and an elevated troponin level (COVID+/troponin+) were enrolled between June 2020 and March 2021 and had a magnetic resonance imaging scan within 28 days of discharge. Two prospective control groups were recruited, comprising 64 patients with COVID-19 and normal troponin levels (COVID+/troponin−) and 113 patients without COVID-19 or elevated troponin level matched by age and cardiovascular comorbidities (COVID−/comorbidity+). Regression modeling was performed to identify predictors of major adverse cardiovascular events at 12 months.

**Results:**

Of the 519 included patients, 356 (69%) were men, with a median (interquartile range) age of 61.0 years (53.8, 68.8). The frequency of any heart abnormality, defined as left or right ventricular impairment, scar, or pericardial disease, was 2-fold greater in cases (61% [207/342]) compared with controls (36% [COVID+/troponin−] versus 31% [COVID−/comorbidity+]; *P*<0.001 for both). More cases than controls had ventricular impairment (17.2% versus 3.1% and 7.1%) or scar (42% versus 7% and 23%; *P*<0.001 for both). The myocardial injury pattern was different, with cases more likely than controls to have infarction (13% versus 2% and 7%; *P*<0.01) or microinfarction (9% versus 0% and 1%; *P*<0.001), but there was no difference in nonischemic scar (13% versus 5% and 14%; *P*=0.10). Using the Lake Louise magnetic resonance imaging criteria, the prevalence of probable recent myocarditis was 6.7% (23/342) in cases compared with 1.7% (2/113) in controls without COVID-19 (*P*=0.045). During follow-up, 4 patients died and 34 experienced a subsequent major adverse cardiovascular event (10.2%), which was similar to controls (6.1%; *P*=0.70). Myocardial scar, but not previous COVID-19 infection or troponin, was an independent predictor of major adverse cardiovascular events (odds ratio, 2.25 [95% CI, 1.12–4.57]; *P*=0.02).

**Conclusions:**

Compared with contemporary controls, patients with COVID-19 and elevated cardiac troponin level have more ventricular impairment and myocardial scar in early convalescence. However, the proportion with myocarditis was low and scar pathogenesis was diverse, including a newly described pattern of microinfarction.

**Registration:**

URL: https://www.isrctn.com; Unique identifier: 58667920.

**Clinical Perspective**

**What Is New?**

- This study is the first large, multicenter, prospective, case-control study investigating the nature and extent of myocardial injury in patients hospitalized with COVID-19 and elevated cardiac troponin level imaged within 28 days of discharge with core laboratory analyses.
- Patients with COVID-19 had a much lower prevalence of probable recent myocarditis than previously reported.
- In patients with COVID-19, we identified a new pattern of microinfarction on cardiac magnetic resonance imaging, highlighting the prothrombotic nature of this disease.
- Among hospitalized patients with COVID-19 and elevated cardiac troponin level, the presence of scar was independently associated with cardiovascular outcomes at 12 months.

**What Are the Clinical Implications?**

- We identified a lower prevalence of probable recent myocarditis than previously described and higher proportions of myocardial infarction and microinfarction in COVID-19. Myocardial scar was independently associated with cardiovascular outcomes. This is important to facilitate appropriate management decisions.

**Editorial, see p 375**

Multiorgan involvement in hospitalized patients with coronavirus disease 2019 (COVID-19) is common, particularly in patients with cardiovascular disease or risk factors, and may result in acute myocardial injury, detected by an increase in cardiac troponin concentrations.^1,2^ Elevated cardiac troponin is associated with a worse prognosis.^2–6^ Multiple mechanisms of myocardial injury have been proposed.^7^ Defining these is important because mitigation or prevention strategies likely depend on the underpinning mechanisms and the sequelae of scar may predispose to late events.

Cardiac magnetic resonance imaging (MRI) is the reference standard for the assessment of acute myocardial injury because it can assess structure, function, scar, and inflammation.^8^ Initial studies of COVID-19 using cardiac MRI reported a myocarditis-like injury pattern was common,^9,10^ but later, more comprehensive reports^11^ also identified myocardial infarction. More recent autopsy studies identified microangiopathic thrombosis as a more common occurrence than myocarditis.^12–14^ However, many of these early imaging studies were necessarily small, were single-center, and used noncontemporaneous or unmatched controls, meaning injury causation and association were not well separated.^10,11,15–17^

COVID-HEART was funded by the National Institute for Health Research and categorized as an urgent public health study in the United Kingdom. The study aims to characterize myocardial injury and its associations and sequelae in convalescent patients after hospitalization with COVID-19 using appropriately matched contemporary controls.

## Methods

### Study Design

The rationale and design of COVID-HEART have been published elsewhere.^18^ Full details of the methods are provided in the Supplemental Material. Briefly, COVID-HEART was a prospective, longitudinal, multicenter, observational cohort study of patients hospitalized with COVID-19 and elevated serum troponin in the United Kingdom, with the aim of characterizing the occurrence, nature, and extent of myocardial injury using cardiac MRI. Anonymized data that support the findings of this study are available from the corresponding author upon reasonable request.

### Study Population

In total, 25 of the 82 cardiac MRI centers in the United Kingdom participated. The study population included patients who were hospitalized with a molecular or clinical and radiologic diagnosis of COVID-19 with evidence of myocardial injury defined as an increase in cardiac troponin I or T above the sex-specific 99th percentile upper reference limit (COVID+/troponin+). Enrollment occurred during the Wuhan and Alpha waves of COVID-19, before vaccination, and when dexamethasone and anticoagulant protocols were emerging. Exclusion criteria were inability or unwillingness to provide consent, contraindication to MRI, pregnancy, or breastfeeding. Two contemporary control populations were prospectively recruited: patients hospitalized with COVID-19 without elevated cardiac troponin (COVID+/troponin−) and an age- and cardiovascular comorbidity–matched community population without COVID-19 (COVID−/comorbidity+). All cases and controls provided written informed consent. Ethical approval was granted by the UK National Research Ethics Service (20/NW/0292) with data sharing under a UK national COPI agreement.^19^ The University of Glasgow Clinical Trials Unit coordinated data and analyses. The study was sponsored by the University of Leeds.

### Cardiac MRI Data Acquisition and Postprocessing

Cardiac MRI scans were performed during index admission or within 28 days of discharge on either a 1.5T or a 3T MRI system (overall 12 scanner models from 3 manufacturers) using protocols consistent with Society for Cardiovascular Magnetic Resonance recommendations for patients with COVID-19.^20^ This included anatomic imaging, long- and short-axis cine scans, and myocardial tissue characterization including T2 mapping, precontrast and postcontrast T1 mapping (for assessment of native T1 and extracellular volume fraction [ECV], where available), and late gadolinium enhancement (LGE; see the Supplemental Material).^18^ MRI data sets were analyzed blinded to disease status within a disseminated core laboratory (Barts: structure/function/LGE; Oxford: T1/T2 mapping; Glasgow: extracardiac anatomy). Left ventricular mass, volumes, and ejection fraction were analyzed using a clinically validated artificial intelligence analysis platform and verified by 2 experts, as were right ventricular volumes and left atrial area.^21^ Impairment was defined as below age- and sex-specific reference ranges. LGE analysis was performed by 3 independent observers blinded to patient details and disease status using a semiautomated signal 5-SD approach (Figure S3). The LGE images were classified visually (consensus of observers with senior author review) into 6 main patterns (infarct: subendocardial or transmural scar conforming to a coronary territory; nonischemic: subepicardial or intramyocardial scar [a category that included most myocarditis-related scars]; infarct and nonischemic: dual pathology; microinfarction: bright subsegmental areas of scar, sometimes in multiple territories; likely preexisting scar or nonspecific scar; or no scar (Figure 2 and Figure S1). Global T1 and T2 mapping were quality-assured (Methods in the Supplemental Material) and analyzed to establish global left ventricular T2, native T1, and ECV values. Results were expressed as normalized values on the basis of normal reference ranges from each center.

**Figure 1.**
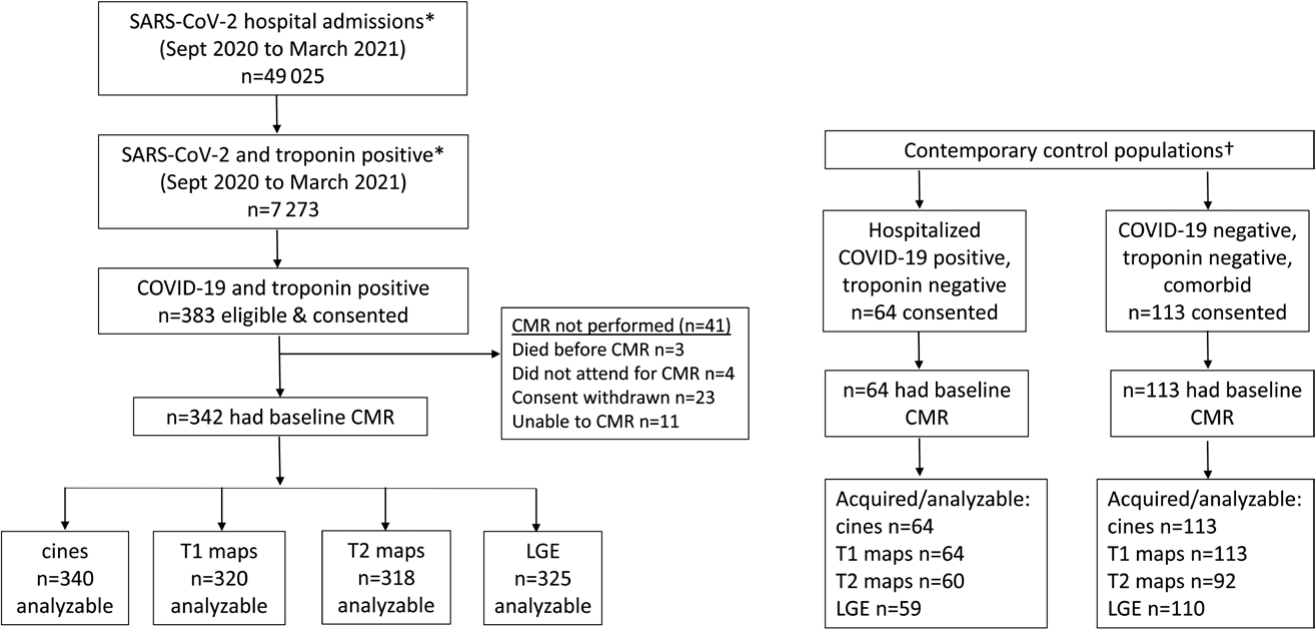
**Study profile.** *From 25 recruiting UK hospitals. †From 5 of the recruiting hospitals. CMR indicates cardiac magnetic resonance imaging; LGE, late gadolinium enhancement; and MRI, magnetic resonance imaging.

### Statistical Analysis

Variables are expressed, as appropriate, as mean±SD, median and interquartile range, or count and percentage. Fisher exact or χ^2^ or test was performed for discrete variables. For continuous variables, comparisons were performed using analysis of variance (ANOVA) or Student *t* test, or by the nonparametric Mann-Whitney test when necessary. When indicated, multiple testing correction was applied using Bonferroni correction. Multivariable logistic regression analysis was performed. Major adverse cardiovascular events (MACEs) were defined as a composite of death, myocardial infarction or acute coronary syndrome, coronary revascularization, myocarditis, hospitalization for other cardiovascular causes, transient ischemic attack, pulmonary embolism, or deep vein thrombosis. The following clinical measures were selected for inclusion in a multivariable logistic regression model: cases versus controls, age, sex, impaired left ventricular ejection fraction (LVEF), and LGE.

IBM-SPSS statistical software version 26 and R software (R Foundation for Statistical Computing) were used for statistical analyses.^18^

## Results

### Study Population

The CONSORT (Consolidated Standards of Reporting Trials) diagram is shown in Figure 1. A total of 49 025 patients with COVID-19 were discharged from the 25 participating hospitals between June 2020 and March 2021 during our enrollment period. Of these, 7273 had an elevated cardiac troponin level, 383 of whom were eligible and provided consent to participate. Cardiac MRI was not performed in 41 patients, with data complete and suitable for analysis in 340 (99%) for cine imaging, 320 (93%) for T1 mapping, 318 (93%) for T2 mapping, and 325 (95%) for LGE (Figure 1).

Demographic and clinical characteristics of participants are shown in Table 1 and Table S1. Patients (COVID+/troponin+) were predominantly male (243/342 [71%]) and 61.3 years of age (53.4, 69.7). Common comorbidities included hypertension (160/342 [47%]), obesity (145/342 [43%]), and diabetes (84/342 [25%]). Cases included current or previous smoking in 36% of patients, 265 (77.5%) received oxygen therapy during hospitalization, 34 (9.9%) required invasive mechanical ventilation, and 190 (56%) received steroid therapy. Sixty-five patients (19.0%) underwent clinically indicated coronary angiography. Of these, 21 (32.3%) had normal coronary arteries, 21 (32.3%) had nonobstructive plaque, and 23 (35.4%) had obstructive plaque (Table S2). At discharge, 128 (34%) received antiplatelet therapy and 134 (35%) received anticoagulation. The working diagnosis for an elevated cardiac troponin level was diverse and was difficult to ascertain in many cases (Table S3). The median length of hospital stay was 9 days (interquartile range, 5 to 16).

**Table 1.**
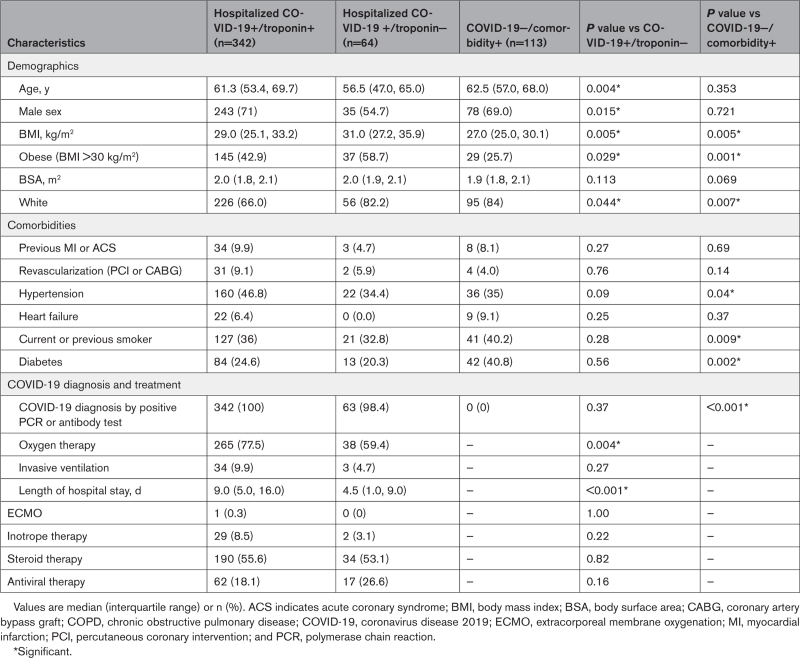
Baseline Characteristics of the Study Population and Control Groups

Controls with COVID-19 who did not have an elevated cardiac troponin level (COVID+/troponin−) were younger (56.5 [47.0, 65.0] years of age) and had a higher body mass index than cases (Table 1). Controls without COVID-19 (COVID−/comorbidity+) were well matched for age, sex, and cardiovascular comorbidities with cases (COVID+/troponin+), but there was some variation in individual risk factors (Table 1). For example, the prevalence of obesity and hypertension was higher in cases, but smoking and diabetes was lower.

### Cardiac MRI Findings

Cardiac assessment was in early convalescence with scan timing at median 21.0 (11.0, 27.0) days after discharge (Table 2).

**Table 2.**
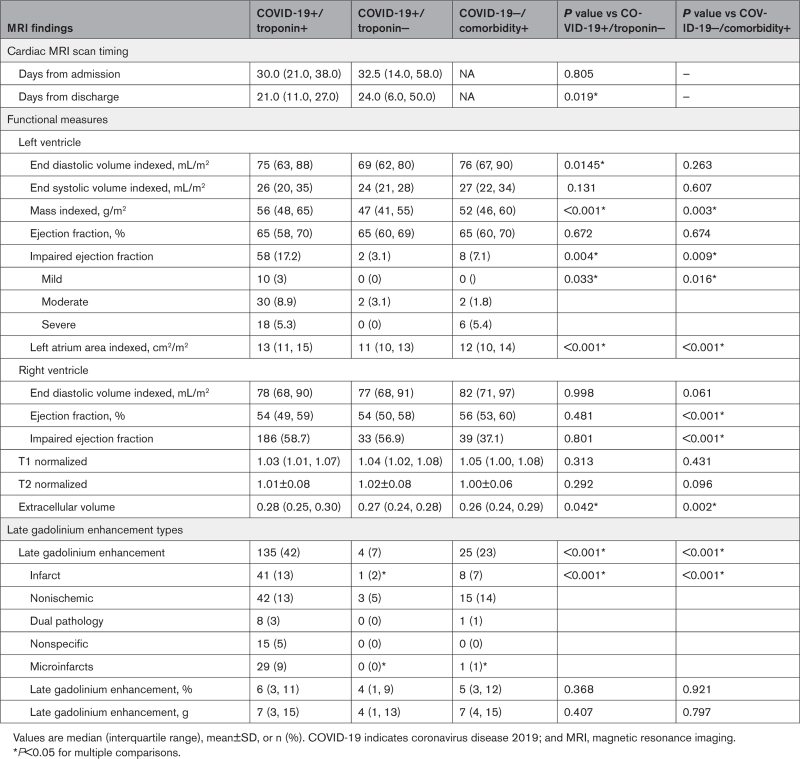
MRI Findings

#### Cardiac Structure and Function

Median LVEF in cases (COVID+/troponin+) was normal and not different from the control populations (65% [58%, 70%] versus 65% [60%, 69%] and 65% [60%, 70%]; *P*=0.67). However, individuals with LV impairment were more frequent among the cases compared with controls. In patients with COVID-19 and elevated cardiac troponin level, LV impairment was categorized as mild (LVEF below age/sex reference range, but >50%) in 3.0%, moderate (LVEF 50% to 40%) in 8.9%, and severe (LVEF <40%) in 5.3% of cases (Table 2). The median LV global longitudinal strain was −15.2 (−17.1, −13.0) in cases and −16.1 (−17.5, −14.5) in controls, but LV mass index was higher in cases, consistent with the increased prevalence of hypertension (Table 2).

The median right ventricular ejection fraction in cases (COVID+/troponin+) was 54% (49%, 59%), and was similar to controls with COVID-19 (54% [50%, 58%]), but was lower than in community controls without COVID-19 (56% [53%, 60%]; *P*<0.001). The right ventricle was impaired in 26.2% of cases, which was comparable to controls with COVID-19 (19.0%) but more frequent than community controls (13.3%; *P*=0.02).

#### Extent and Patterns of Myocardial Injury

**Figure 2.**
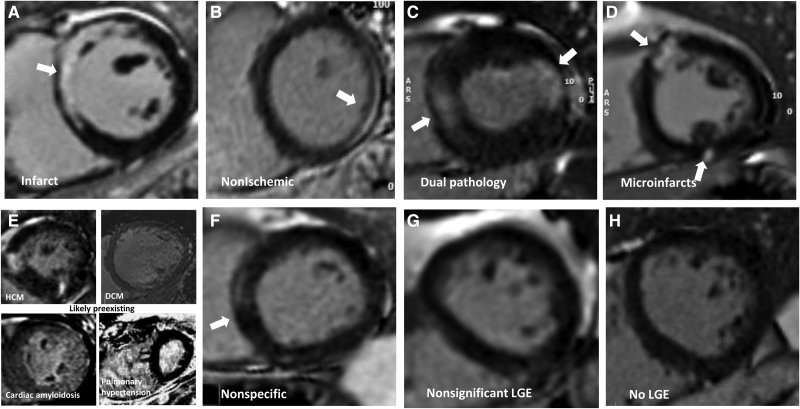
**Patterns of myocardial scar (late gadolinium enhancement).** Patterns of late gadolinium enhancement (LGE; in parentheses, the features of each). **A**, Infarct (bright, subendocardial, or territorial). **B**, Nonischemic (mid-myocardial, less bright, or more diffuse). **C**, Dual pathology (both **A** and **B**). **D**, Microinfarcts (bright spots [eg, approximately 1 gram] of LGE often but not exclusively subendocardial and potentially in >1 territory). **E**, Chronic, likely preexisting disease (only 4 cases total; top left: HCM; top right: dilated cardiomyopathy [DCM]; bottom left: amyloidosis; bottom right: pulmonary hypertension). **F**, Nonspecific (unequivocal LGE that cannot be considered normal and has insufficient volume to assign with certainty to any other category). **G**, Nonsignificant LGE (minor right ventricle insertion point LGE alone; trabecular LGE alone; or septal perforator LGE alone, which can be considered normal variant). **H**, No LGE. Other examples are shown in Figures S1 and S2.

**Figure 3.**
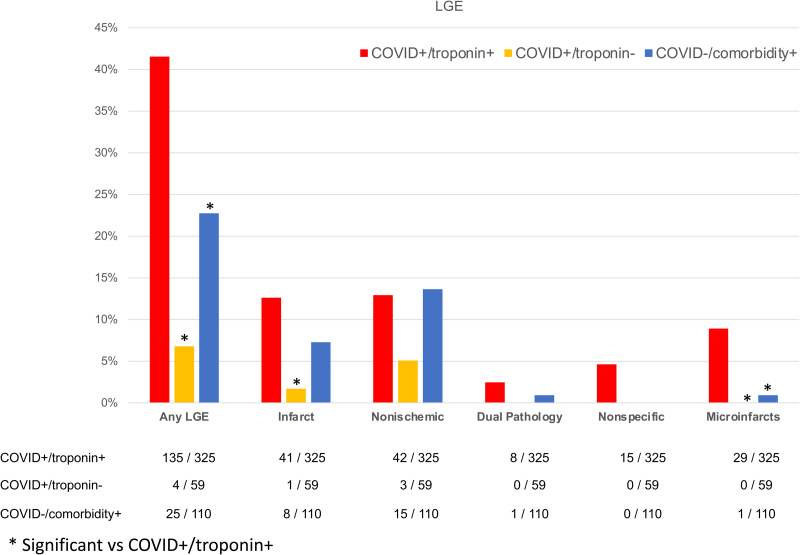
**Late gadolinium enhancement patterns in each cohort.** LGE indicates late gadolinium enhancement.

There were 135 (42%) cases (COVID+/troponin+) with scar on LGE imaging, which was 6-fold higher than controls with COVID-19 (7%; *P*<0.001) and double that of community controls without COVID-19 (23%; *P*<0.001). Where present, the extent of scar was variable and not different between cases and community controls (7 g [3 g, 15 g] and 6% [3%, 11%] versus 7 g [4 g, 15 g] and 5% [3%, 12%]; Table 2). The prevalence of scar in the controls with COVID-19 and normal cardiac troponin concentrations was insufficient for statistical comparison. Scar extent in the cases and community controls was associated with adverse cardiac remodeling (ventricular or atrial dilatation, hypertrophy, impairment; Table S4) and patients with LV impairment had a higher frequency and extent of scar (Figure S5). Scar pattern classifications are shown in Figure 2 with multiple examples in Figures S1 and S2. Cases had a higher frequency of myocardial infarction (13% versus 2%) and microinfarction (9% versus 1%) pattern scar than controls; this latter pattern was almost exclusively found in patients with COVID-19 and elevated cardiac troponin. Examples of the microinfarction pattern are shown in Figure 2. The prevalence of other scar patterns (nonischemic including myocarditis, dual pathology, and nonspecific) were not different between cases and the control groups (Figure 3).

### Myocardial Tissue Characterization (Mapping)

Mapping results are shown in Table 2 and Figure S4. Normalized global T1 was elevated in cases and both control groups (1.03 [1.01, 1.07], 1.04 [1.02, 1.08], 1.05 [1.00,1.08] versus a normal of 1.00; *P*<0.001), but there were no between-group differences. The myocardial extracellular volume was elevated (~1%) in cases compared with both control groups (0.28 [0.25, 0.30] versus 0.27 [0.24, 0.28], *P*=0.04, and 0.26 [0.24, 0.29], *P*=0.002, respectively). Global myocardial T2 values were no different across groups and were not elevated (Table 2). In cases, where T2 values were measurable (n=102), T2 was higher in LGE areas compared with remote myocardium (∆T2 +5.1 ms). Infarct and microinfarct patterns had the highest ∆T2: +8.6 ms compared with +5.2 ms for mixed and +3.5 ms for nonischemic patterns.

### Cardiac Troponin Concentrations

The peak cardiac troponin concentration was higher in cases with evidence of LGE compared with cases without LGE (14.2 versus 3.7 × upper limit of normal, *P*<0.01; TnT 3.0 versus 2.1 × upper limit of normal, *P*=0.04). Cases with myocardial infarction and microinfarction patterns had higher cardiac troponin values than those with no LGE (both *P*<0.05), with other LGE patterns comparable to that of no LGE (Figure S6). The magnitude of the troponin elevation was not related to respiratory support status (*P*=NS).

### Patient-Level Excess Cardiac Pathology

The frequency of any heart abnormality defined as left or right ventricular impairment, scar, or pericardial disease was 2-fold greater in cases (61% [207/342]) compared with controls (36% [COVID+/troponin−] and 31% [COVID−/comorbidity+]; *P*<0.001 for both). This was driven by more LV and right ventricle impairment (5% and 15%, both associated with more scar, the excess scar being exclusively infarction and microinfarction) and 10% more pericardial effusions but no extra scar in those without ventricular impairment (Figure S7).

We applied the updated 2018 Lake Louise MRI criteria to identify probable recent myopericarditis.^22^ There was no overall global T2 elevation and T1 elevation was comparable across cases and both control groups, but there was some evidence of myocardial scar in a pattern consistent with recent myocarditis. This was found in the nonischemic category (n=42), the nonischemic component of the dual pathology group (n=8), and in the low volume nonspecific scar group without known preexisting disease (n=11). The overall frequency of this scar pattern was not different in cases compared with control groups. We sought evidence that this scar could be recent, with either T2 elevation (>5 ms) or a pericardial effusion (ie, probable recent myocarditis). Comparing cases and community controls, the frequency was 37% (23/61) and 13% (2/16), respectively (*P*=0.06). This resulted in an overall prevalence of myocarditis of 6.7% (23/342) in cases compared with 1.7% (2/113) in community controls, an excess of 5% overall (*P*=0.045).

### Cardiac Measures and Disease Severity

In cases, there was no association between cardiac LV/right ventricle structure and function and respiratory support requirements. However, scar was more prevalent in those who received no or oxygen-only respiratory support (Table S5).

### Clinical Follow-Up

At 12 months, 4 (1.2%) patients with COVID-19 and an elevated cardiac troponin level and 2 (1.2%) community controls without COVID-19 had died. MACE was reported in 34 (10.2%) cases and in 10 (6.1%) persons from the combined control groups (Table 3). In detail, comparing cases with the combined control group, 4 (1.2%) and 2 (1.2%) patients experienced myocardial infarction or acute coronary syndrome and coronary revascularization was performed in 4 (1.2%) and 1 (0.6%) patients, respectively. Myocarditis was reported in 10 (3%) and 0 (0%) patients, and pericarditis in 1 (0.3%) and 0 (0%) patients, respectively, with 13 (3.9%) and 1 (0.6%) hospitalized for other cardiovascular causes.

**Table 3.**
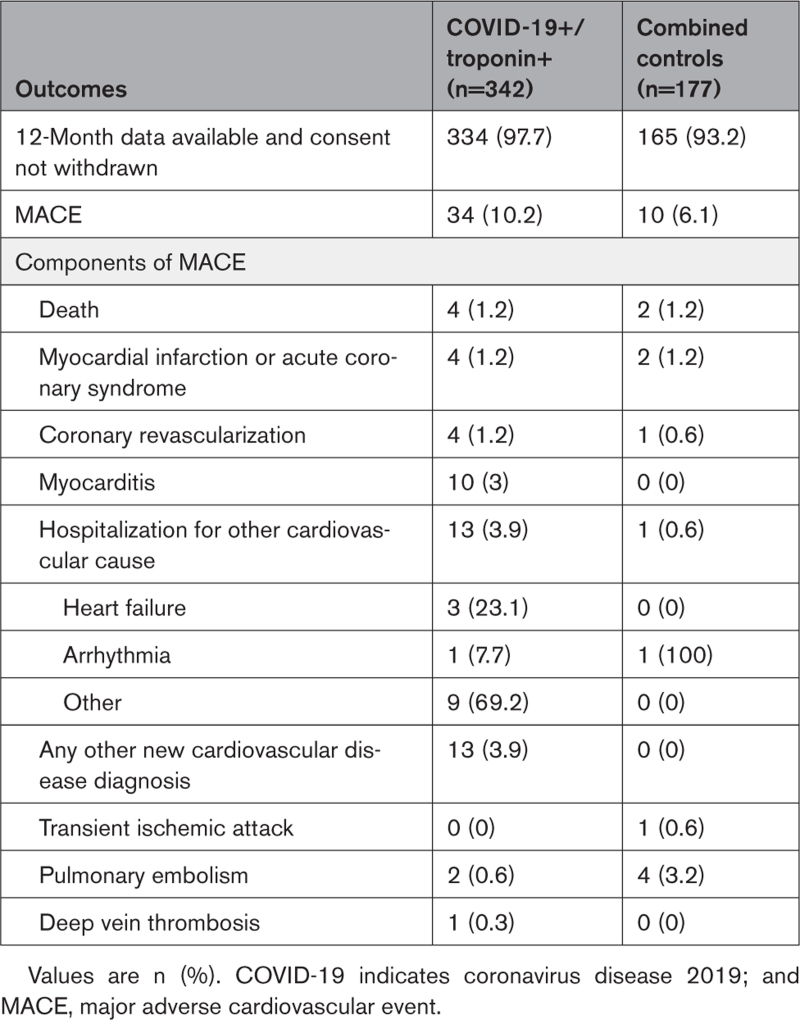
Clinical Outcomes at 12-Month Follow-Up

By multivariable logistic regression, including age, sex, LV dysfunction, and LGE, the presence of LGE was associated with MACE (odds ratio, 2.25 [95% CI, 1.12–4.57]; *P*=0.02). Conversely, impaired LVEF or previous COVID-19 infection/troponin status were not associated with MACE (odds ratio, 1.54 [95% CI, 0.64–3.49]; *P*=0.23; odds ratio, 1.17 [95% CI, 0.54–2.67]; *P*=0.70; Table 4).

**Table 4.**
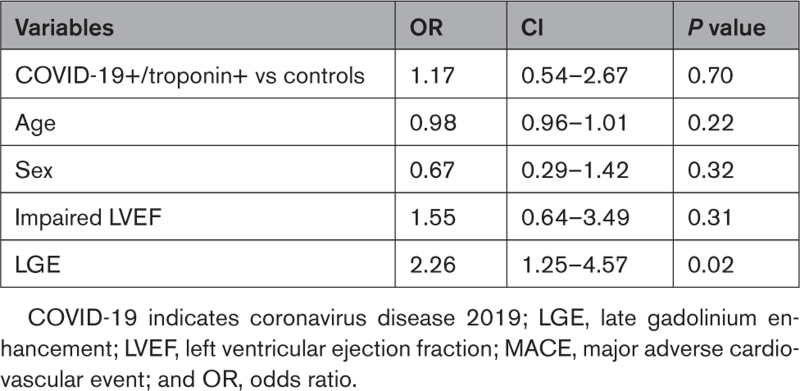
Multivariable Analysis of MACE Risk at 12-Month Follow-Up

## DISCUSSION

This prospective, multicenter, case–control study explored how COVID-19 can affect the heart, focusing on hospitalized patients with relatively severe COVID-19 and elevated cardiac troponin levels. The working clinical diagnosis for myocardial injury was diverse and difficult to ascertain in many cases. This highlights the challenge of defining the mechanism of myocardial injury in COVID-19 without the aid of MRI. Cases were compared with 2 control groups that were scanned contemporaneously: patients hospitalized with COVID-19 but normal cardiac troponin concentrations and community controls without COVID-19 matched by age, sex, and cardiovascular comorbidity. This enabled us to differentiate the effects of COVID-19 infection from previous myocardial scarring and injury attributable to cardiovascular comorbidities. Our main finding was that patients with COVID-19 and myocardial injury were twice as likely to have cardiac abnormalities than either control population. This included more patients with left and right ventricular systolic impairment, more pericardial effusions, and more myocardial scarring. The scar pattern suggested the pathogenesis of injury was different from that of controls. Although there was some evidence of probable recent myocarditis in 1 in 20 cases, excess scar was predominantly from myocardial infarction and microinfarction, highlighting the prothrombotic sequelae of COVID-19.

Myocardial scar was not uncommon in both control populations (7% and 23%), with a variety of patterns reflecting the high prevalence of comorbidities, which may lead clinicians to underestimate preexisting scar in multimorbid patients^23,24^ and therefore falsely attribute scar to the intercurrent COVID-19 infection episode (case ascertainment bias). The association of scar with LV hypertrophy and impairment within just 28 days from discharge further supports the hypothesis that at least a proportion of LGE represents preexisting scar.

The large size of this study permitted more granular adjudication of the scar pattern than previous,

smaller studies^4,11,16^ and the focus on hospitalized patients with COVID-19 and elevated cardiac troponin concentrations meant abnormalities were expected to be common. Our understanding of scar patterns comes from previous research showing that myocardial infarction has a specific pattern (subendocardial, bright, territorial), whereas nonischemic scar from a variety of different pathogens or insults typically has a mid-myocardial, often more lateral wall pattern.

For a new disease, caution is needed in interpretation with respect to case ascertainment bias. In this study, the excess scar appeared to be predominantly infarction and microinfarction, the latter a pattern that appeared near-specific to patients with COVID-19 and elevated cardiac troponin level compared with controls. These small, often multiterritory, bright subsegmental areas of scar are familiar from studies evaluating patients with acute myocardial injury after coronary angioplasty.^25^ The simplest explanation is that a substantial component of COVID-19–related myocardial injury appears related to macroangiopathic and microangiopathic thrombosis caused by a prothrombotic state, a phenomenon now familiar in multiple vascular beds during the inflammatory phase of infection. Cardiac troponin elevations were higher in patients with infarct and microinfarct patterns, strengthening the hypothesis of cardiac microangiopathic and macroangiopathic thrombosis.

At 12-month follow-up, only 4 (1.2%) patients with COVID-19 and elevated cardiac troponin had died, and 34 (10%) had experienced MACE. In line with other cardiac pathologies,^26–29^ the presence of LGE had the strongest association with clinical outcomes and this should be considered in the management of hospitalized patients with COVID-19 with troponin elevation. Despite cases seeming to have more clinical events than controls, after adjustment for other variables, this was no longer statistically significant; this finding is in keeping with a recent, very large population study.^30^ This raises the question of whether it is the presence of scar, rather than the COVID-19 status per se, that worsens the cardiovascular prognosis.

Although our study is affected by survivor bias, and despite the potential for myocarditis to heal before cardiac MRI, it provides some evidence to support COVID-19 myocarditis and direct myotropic injury, although this was found in excess in only 1 in 20 cases compared with controls, even among a cohort of hospitalized patients with relatively severe illness and elevated cardiac troponin level. These observations are consistent with several case reports^31,32^ and with the most recent cardiac autopsy meta-analysis of 22 studies, in which there is a reducing focus on myocarditis (7.2% prevalence but only 1.4% functionally significant) and more focus on microangiopathic thrombosis,^33,34^ with one study highlighting these as common, occurring even without epicardial coronary artery disease, and being widely distributed at all levels of the vascular tree.^35^ This prothrombotic state may arise from hypercoagulability, endothelial injury, vasculitis, cytokine storm, and thrombosis itself,^36^ with pulmonary embolism, strokes, small asymptomatic brain infarcts, and peripheral embolism being common.^33,37,38^

Myocardial scar is clearly important. It has functional consequences,^39^ even before overt LV impairment, and is associated with adverse outcome in almost all diseases studied to date.^40,41^ Myocardial scar was associated with reduced LV ejection fraction in all 3 groups. As with other diseases, we suspect myocardial scar after COVID-19 may be a marker of future vulnerability to subsequent insults and an adverse prognostic marker.

Myocardial inflammation was also investigated. Overall, the MRI mapping results are clinically reassuring. Whereas T2 was elevated in scar areas, supporting the concept of acute myocardial injury, there was no generalized elevation compared with our control patients. T1 and ECV values were mildly elevated, as others have found in COVID-19 and in those with comorbidities,^39^ but not more so in the cases compared with both control groups. These data corroborate recent reports^42^ that persisting cardiac inflammation is not as high as initial reports suggested, refocusing attention on thrombosis (here infarction and microinfarction) and deemphasizing myocarditis.

Our findings suggest that respiratory disease severity during hospitalization does not reflect cardiac disease severity. Although 1-third of patients with COVID-19 and elevated cardiac troponin had residual lung changes reflecting the hospital illness severity, myocardial scar was not more prevalent in those with severe lung involvement. This may reflect a survivor bias (with ventilated patients with troponin elevations not surviving) or intensive care troponin release being diffuse without focal scar, or it may represent fewer patients having had bystander infection, which may be more likely in those with an acute cardiac presentation.

There are several strengths and limitations that merit consideration. The study was designed to look only at those patients with COVID-19 who sustained an elevation in cardiac troponin during their hospital admission and survived. Subsequent MRI at 6 months and effect of recovery status is pending. Study strengths are that this is a national prospective study with clearly defined inclusion criteria, standardized imaging protocol, blinded core laboratory analyses, and independent statistical analyses by a certified clinical trials unit. However, no patients had baseline prepandemic MRI scans; therefore, it is possible that patients with preexisting cardiac abnormalities (LV impairment, scar) might be more likely to be hospitalized with COVID-19 or have an elevated cardiac troponin level as a response. We only recruited patients with an elevated cardiac troponin level for whom this was measured as part of routine clinical care and, therefore, we cannot comment on the true prevalence of elevated cardiac troponin level in this population. There may also have been some patients who had asymptomatic COVID-19 identified on screening with polymerase chain reaction. Sites used their local MRI scanning procedures following a prespecified imaging protocol with harmonization performed post hoc. MRI tissue mapping was up to 3 slices and not whole heart. Because of the relatively low number of events, the combined end point was very broad.

Convalescing patients with COVID-19 who had acute myocardial injury during their hospitalization were twice as likely to have abnormalities on cardiac MRI compared with matched contemporary controls. These patients had more ventricular impairment and myocardial scar than those with COVID-19 who did not have elevated cardiac troponin concentrations and community controls without COVID-19 who were matched for cardiovascular comorbidities. Excess scar was typically that of myocardial infarction or microinfarction, the latter being a newly described imaging pattern, with myocardial inflammation and probable recent myocarditis occurring less frequently. These findings suggest that macroangiopathic and microangiopathic thrombosis may be the key pathologic process for myocardial injury in COVID-19 survivors. Future work will determine the prognostic, functional, and quality of life effects of these changes in the longer term.

## Article Information

### Acknowledgments

Dr Berry acknowledges British Heart Foundation support (grant RE/18/6134217). Dr Artico received funding from the European Association of Cardiovascular Imaging (EACVI research grant App000073878). Dr McCann is funded by an NIHR research professorship (RP-2017-08-ST2-007). Dr Manisty is funded by an NIHR clinician scientist award (CS-2015-15-003). Drs Ferreira, Piechnik, and Neubauer thank the NIHR Oxford Biomedical Research Centre for support of this study. Dr Bucciarelli-Ducci is supported in part by the NIHR Biomedical Research Centre at University Hospitals Bristol National Health Service Foundation Trust and the University of Bristol. Additional support was provided by the NIHR Leicester Biomedical Research Centre and the NIHR Leeds Clinical Research Facility. Dr Dweck is supported by the British Heart Foundation (grant FS/SCRF/21/32010). The authors thank the patients and staff who supported this project.

### Sources of Funding

Supported by NIHR and UK Research and Innovation (COV0254). West Yorkshire and Humber Clinical Research Network (CV070) funded patient information leaflet translation.

### Disclosures

Dr Berry is employed by the University of Glasgow, which holds research or consultancy agreements with AstraZeneca, Abbott Vascular, Boehringer Ingelheim, GSK, HeartFlow, Opsens, and Novartis. Dr Miller has served on advisory boards for Novartis, Boehringer Ingelheim, Lilly Alliance, and AstraZeneca; serves as an advisor for HAYA Therapeutics and PureTech Health; and has received research support from Amicus Therapeutics, Guerbet Laboratories Limited, Roche, and Univar Solutions BV. Dr Moon has served on advisory boards for Sanofi and Genzyme. Dr Bucciarelli-Ducci is the chief executive officer (part time) of the Society for Magnetic Resonance. The other authors have no conflicts to declare.

### Supplemental Material

List of investigators

Expanded Methods, including cardiac magnetic resonance imaging protocol and analysis

Figures S1–S7

Tables S1–S6

## Supplementary Material

## References

[1] Dweck MR Bularga A Hahn RT Bing R Lee KK Chapman AR White A Salvo GD Sade LE Pearce K. Global evaluation of echocardiography in patients with COVID-19. Eur Heart J Cardiovasc Imaging. 2020;21:949–958. doi: 10.1093/ehjci/jeaa178 32556199 10.1093/ehjci/jeaa178 PMC7337658

[2] Giustino G Croft LB Stefanini GG Bragato R Silbiger JJ Vicenzi M Danilov T Kukar N Shaban N Kini A. Characterization of myocardial injury in patients with COVID-19. J Am Coll Cardiol. 2020;76:2043–2055. doi: 10.1016/j.jacc.2020.08.069 33121710 10.1016/j.jacc.2020.08.069 PMC7588179

[3] Sengupta PP Chandrashekhar YS. Cardiac involvement in the COVID-19 pandemic: hazy lessons from cardiac imaging? JACC Cardiovasc Imaging. 2020;13:2480–2483. doi: 10.1016/j.jcmg.2020.10.001 33153538 10.1016/j.jcmg.2020.10.001 PMC7547566

[4] Knight DS Kotecha T Razvi Y Chacko L Brown JT Jeetley PS Goldring J Jacobs M Lamb LE Negus R. COVID-19: myocardial injury in survivors. Circulation. 2020;142:1120–1122. doi: 10.1161/CIRCULATIONAHA.120.049252 32673505 10.1161/CIRCULATIONAHA.120.049252 PMC7492396

[5] Nie SF Yu M Xie T Yang F Wang HB Wang ZH Li M Gao XL Lv BJ Wang SJ. Cardiac troponin I is an independent predictor for mortality in hospitalized patients with COVID-19. Circulation. 2020;142:608–610. doi: 10.1161/CIRCULATIONAHA.120.048789 32539541 10.1161/CIRCULATIONAHA.120.048789 PMC7418761

[6] Karbalai Saleh S Oraii A Soleimani A Hadadi A Shajari Z Montazeri M Moradi H Talebpour M Sadat Naseri A Balali P. The association between cardiac injury and outcomes in hospitalized patients with COVID-19. Intern Emerg Med. 2020;15:1415–1424. doi: 10.1007/s11739-020-02466-1 32772283 10.1007/s11739-020-02466-1 PMC7415198

[7] Sandoval Y Januzzi JL Jr Jaffe AS. Cardiac troponin for assessment of myocardial injury in COVID-19: JACC review topic of the week. J Am Coll Cardiol. 2020;76:1244–1258. doi: 10.1016/j.jacc.2020.06.068 32652195 10.1016/j.jacc.2020.06.068 PMC7833921

[8] Leiner T Bogaert J Friedrich MG Mohiaddin R Muthurangu V Myerson S Powell AJ Raman SV Pennell DJ. SCMR position paper (2020) on clinical indications for cardiovascular magnetic resonance. J Cardiovasc Magn Reson. 2020;22:76. doi: 10.1186/s12968-020-00682-4 33161900 10.1186/s12968-020-00682-4 PMC7649060

[9] Nagel E Puntmann VO. Errors in statistical numbers and data in study of cardiovascular magnetic resonance imaging in patients recently recovered from COVID-19. JAMA Cardiol. 2020;5:1307–1308. doi: 10.1001/jamacardio.2020.4661 32840561 10.1001/jamacardio.2020.4661

[10] Puntmann VO Carerj ML Wieters I Fahim M Arendt C Hoffmann J Shchendrygina A Escher F Vasa-Nicotera M Zeiher AM. Outcomes of cardiovascular magnetic resonance imaging in patients recently recovered from coronavirus disease 2019 (COVID-19). JAMA Cardiol. 2020;5:1265–1273. doi: 10.1001/jamacardio.2020.3557 32730619 10.1001/jamacardio.2020.3557 PMC7385689

[11] Kotecha T Knight DS Razvi Y Kumar K Vimalesvaran K Thornton G Patel R Chacko L Brown JT Coyle C. Patterns of myocardial injury in recovered troponin-positive COVID-19 patients assessed by cardiovascular magnetic resonance. Eur Heart J. 2021;42:1866–1878. doi: 10.1093/eurheartj/ehab075 33596594 10.1093/eurheartj/ehab075 PMC7928984

[12] Bussani R Schneider E Zentilin L Collesi C Ali H Braga L Volpe MC Colliva A Zanconati F Berlot G. Persistence of viral RNA, pneumocyte syncytia and thrombosis are hallmarks of advanced COVID-19 pathology. EBioMedicine. 2020;61:103104. doi: 10.1016/j.ebiom.2020.103104 33158808 10.1016/j.ebiom.2020.103104 PMC7677597

[13] Kawakami R Sakamoto A Kawai K Gianatti A Pellegrini D Nasr A Kutys B Guo L Cornelissen A Mori M. Pathological evidence for SARS-CoV-2 as a cause of myocarditis: JACC review topic of the week. J Am Coll Cardiol. 2021;77:314–325. doi: 10.1016/j.jacc.2020.11.031 33478655 10.1016/j.jacc.2020.11.031 PMC7816957

[14] CAPACITY-COVID Collaborative Consortium and LEOSS Study Group Clinical presentation, disease course, and outcome of COVID-19 in hospitalized patients with and without pre-existing cardiac disease: a cohort study across 18 countries. Eur Heart J. 2021;43:1104–1120. doi: 10.1093/eurheartj/ehab656 10.1093/eurheartj/ehab656 34734634

[15] Esposito A Palmisano A Natale L Ligabue G Peretto G Lovato L Vignale D Fiocchi F Marano R Russo V. Cardiac magnetic resonance characterization of myocarditis-like acute cardiac syndrome in COVID-19. JACC Cardiovasc Imaging. 2020;13:2462–2465. doi: 10.1016/j.jcmg.2020.06.003 32654966 10.1016/j.jcmg.2020.06.003 PMC7314439

[16] Ng MY Ferreira VM Leung ST Yin Lee JC Ho-Tung Fong A To Liu RW Man Chan JW Wu AKL Lung KC Crean AM. Patients recovered from COVID-19 show ongoing subclinical myocarditis as revealed by cardiac magnetic resonance imaging. JACC Cardiovasc Imaging. 2020;13:2476–2478. doi: 10.1016/j.jcmg.2020.08.012 33153536 10.1016/j.jcmg.2020.08.012 PMC7455163

[17] Huang L Zhao P Tang D Zhu T Han R Zhan C Liu W Zeng H Tao Q Xia L. Cardiac involvement in patients recovered from COVID-2019 identified using magnetic resonance imaging. JACC Cardiovasc Imaging. 2020;13:2330–2339. doi: 10.1016/j.jcmg.2020.05.004 32763118 10.1016/j.jcmg.2020.05.004 PMC7214335

[18] Gorecka M McCann GP Berry C Ferreira VM Moon JC Miller CA Chiribiri A Prasad S Dweck MR Bucciarelli-Ducci C; COVID-HEART investigators Demographic, multi-morbidity and genetic impact on myocardial involvement and its recovery from COVID-19: protocol design of COVID-HEART-a UK, multicentre, observational study. J Cardiovasc Magn Reson. 2021;23:77. doi: 10.1186/s12968-021-00752-1 34112195 10.1186/s12968-021-00752-1 PMC8190746

[19] NHS Digital. Control of Patient Information (COPI) notice. Published 2022 https://digital.nhs.uk/coronavirus/coronavirus-covid-19-response-information-governance-hub/control-of-patient-information-copi-notice

[20] Kelle S Bucciarelli-Ducci C Judd RM Kwong RY Simonetti O Plein S Raimondi F Weinsaft JW Wong TC Carr J. Society for Cardiovascular Magnetic Resonance (SCMR) recommended CMR protocols for scanning patients with active or convalescent phase COVID-19 infection. J Cardiovasc Magn Reson. 2020;22:61. doi: 10.1186/s12968-020-00656-6 32878639 10.1186/s12968-020-00656-6 PMC7467754

[21] Augusto JB Davies RH Bhuva AN Knott KD Seraphim A Alfarih M Lau C Hughes RK Lopes LR Shiwani H. Diagnosis and risk stratification in hypertrophic cardiomyopathy using machine learning wall thickness measurement: a comparison with human test-retest performance. Lancet Digital Health. 2021;3:e20–e28. doi: 10.1016/S2589-7500(20)30267-3 33735065 10.1016/S2589-7500(20)30267-3

[22] Ferreira VM Schulz-Menger J Holmvang G Kramer CM Carbone I Sechtem U Kindermann I Gutberlet M Cooper LT Liu P. Cardiovascular magnetic resonance in nonischemic myocardial inflammation: expert recommendations. J Am Coll Cardiol. 2018;72:3158–3176. doi: 10.1016/j.jacc.2018.09.072 30545455 10.1016/j.jacc.2018.09.072

[23] Shanbhag SM Greve AM Aspelund T Schelbert EB Cao JJ Danielsen R Thornorgeirsson G Sigurethsson S Eiriksdottir G Harris TB. Prevalence and prognosis of ischaemic and non-ischaemic myocardial fibrosis in older adults. Eur Heart J. 2019;40:529–538. doi: 10.1093/eurheartj/ehy713 30445559 10.1093/eurheartj/ehy713 PMC6657269

[24] Kwong RY Sattar H Wu H Vorobiof G Gandla V Steel K Siu S Brown KA. Incidence and prognostic implication of unrecognized myocardial scar characterized by cardiac magnetic resonance in diabetic patients without clinical evidence of myocardial infarction. Circulation. 2008;118:1011–1020. doi: 10.1161/CIRCULATIONAHA.107.727826 18725488 10.1161/CIRCULATIONAHA.107.727826 PMC2743310

[25] Ricciardi MJ Wu E Davidson CJ Choi KM Klocke FJ Bonow RO Judd RM Kim RJ. Visualization of discrete microinfarction after percutaneous coronary intervention associated with mild creatine kinase-MB elevation. Circulation. 2001;103:2780–2783. doi: 10.1161/hc2301.092121 11401931 10.1161/hc2301.092121

[26] Musa TA Treibel TA Vassiliou VS Captur G Singh A Chin C Dobson LE Pica S Loudon M Malley T. Myocardial scar and mortality in severe aortic stenosis. Circulation. 2018;138:1935–1947. doi: 10.1161/CIRCULATIONAHA.117.032839 30002099 10.1161/CIRCULATIONAHA.117.032839 PMC6221382

[27] Alba AC Gaztanaga J Foroutan F Thavendiranathan P Merlo M Alonso-Rodriguez D Vallejo-Garcia V Vidal-Perez R Corros-Vicente C Barreiro-Perez M. Prognostic value of late gadolinium enhancement for the prediction of cardiovascular outcomes in dilated cardiomyopathy: an international, multi-institutional study of the MINICOR Group. Circ Cardiovasc Imaging. 2020;13:e010105. doi: 10.1161/CIRCIMAGING.119.010105 32312112 10.1161/CIRCIMAGING.119.010105

[28] Dang Y Hou Y. The prognostic value of late gadolinium enhancement in heart diseases: an umbrella review of meta-analyses of observational studies. Eur Radiol. 2021;31:4528–4537. doi: 10.1007/s00330-020-07437-w 33409800 10.1007/s00330-020-07437-w

[29] Becker MAJ Cornel JH van de Ven PM van Rossum AC Allaart CP Germans T. The prognostic value of late gadolinium-enhanced cardiac magnetic resonance imaging in nonischemic dilated cardiomyopathy: a review and meta-analysis. JACC Cardiovasc Imaging. 2018;11:1274–1284. doi: 10.1016/j.jcmg.2018.03.006 29680351 10.1016/j.jcmg.2018.03.006

[30] Rezel-Potts E Douiri A Sun X Chowienczyk PJ Shah AM Gulliford MC. Cardiometabolic outcomes up to 12 months after COVID-19 infection: a matched cohort study in the UK. PLoS Med. 2022;19:e1004052. doi: 10.1371/journal.pmed.1004052 35853019 10.1371/journal.pmed.1004052 PMC9295991

[31] Gorecka M Thirunavukarasu S Levelt E Greenwood JP. Multiple etiologies to myocardial injury in COVID-19. JACC Case Rep. 2021;3:971–972. doi: 10.1016/j.jaccas.2021.05.003 34179834 10.1016/j.jaccas.2021.05.003 PMC8208893

[32] Bhandari SS Yeo J Kotecha D McCann GP. Fulminant micro and macroangiopathic sequalae in a patient with COVID-19. Eur Heart J Case Rep. 2020;4:1–2. doi: 10.1093/ehjcr/ytaa372 10.1093/ehjcr/ytaa372 PMC7717199 33623857

[33] McFadyen JD Stevens H Peter K. The emerging threat of (micro)thrombosis in COVID-19 and its therapeutic implications. Circ Res. 2020;127:571–587. doi: 10.1161/CIRCRESAHA.120.317447 32586214 10.1161/CIRCRESAHA.120.317447 PMC7386875

[34] Halushka MK Vander Heide RS. Myocarditis is rare in COVID-19 autopsies: cardiovascular findings across 277 postmortem examinations. Cardiovasc Pathol. 2021;50:107300. doi: 10.1016/j.carpath.2020.107300 33132119 10.1016/j.carpath.2020.107300 PMC7583586

[35] Pellegrini D Kawakami R Guagliumi G Sakamoto A Kawai K Gianatti A Nasr A Kutys R Guo L Cornelissen A. Microthrombi as a major cause of cardiac injury in COVID-19: a pathologic study. Circulation. 2021;143:1031–1042. doi: 10.1161/CIRCULATIONAHA.120.051828 33480806 10.1161/CIRCULATIONAHA.120.051828

[36] Spence JD de Freitas GR Pettigrew LC Ay H Liebeskind DS Kase CS Del Brutto OH Hankey GJ Venketasubramanian N. Mechanisms of stroke in COVID-19. Cerebrovasc Dis. 2020;49:451–458. doi: 10.1159/000509581 32690850 10.1159/000509581 PMC7445374

[37] Ellul MA Benjamin L Singh B Lant S Michael BD Easton A Kneen R Defres S Sejvar J Solomon T. Neurological associations of COVID-19. Lancet Neurol. 2020;19:767–783. doi: 10.1016/S1474-4422(20)30221-0 32622375 10.1016/S1474-4422(20)30221-0 PMC7332267

[38] Helms J Kremer S Merdji H Clere-Jehl R Schenck M Kummerlen C Collange O Boulay C Fafi-Kremer S Ohana M. Neurologic features in severe SARS-CoV-2 infection. N Engl J Med. 2020;382:2268–2270. doi: 10.1056/NEJMc2008597 32294339 10.1056/NEJMc2008597 PMC7179967

[39] Li X Wang H Zhao R Wang T Zhu Y Qian Y Liu B Yu Y Han Y. Elevated extracellular volume fraction and reduced global longitudinal strains in participants recovered from COVID-19 without clinical cardiac findings. Radiology. 2021;299:E230–E240. doi: 10.1148/radiol.2021203998 33434112 10.1148/radiol.2021203998 PMC7808090

[40] Zemrak F Petersen SE. Late gadolinium enhancement CMR predicts adverse cardiovascular outcomes and mortality in patients with coronary artery disease: systematic review and meta-analysis. Prog Cardiovasc Dis. 2011;54:215–229. doi: 10.1016/j.pcad.2011.07.003 22014489 10.1016/j.pcad.2011.07.003

[41] Grigoratos C Barison A Ivanov A Andreini D Amzulescu MS Mazurkiewicz L De Luca A Grzybowski J Masci PG Marczak M. Meta-analysis of the prognostic role of late gadolinium enhancement and global systolic impairment in left ventricular noncompaction. JACC Cardiovasc Imaging. 2019;12:2141–2151. doi: 10.1016/j.jcmg.2018.12.029 30878415 10.1016/j.jcmg.2018.12.029

[42] Ammirati E Lupi L Palazzini M Hendren NS Grodin JL Cannistraci CV Schmidt M Hekimian G Peretto G Bochaton T. Prevalence, characteristics, and outcomes of COVID-19-associated acute myocarditis. Circulation. 2022;145:1123–1139. doi: 10.1161/CIRCULATIONAHA.121.056817 35404682 10.1161/CIRCULATIONAHA.121.056817 PMC8989611

