# Supplementary material for: Myocardial Involvement After Hospitalization for COVID-19 Complicated by Troponin Elevation: A Prospective, Multicenter, Observational Study
Source: Circulation. 2023 Jan 31;147(5):364–74. doi: 10.1161/CIRCULATIONAHA.122.060632 (PMC9889203; doi:10.1161/CIRCULATIONAHA.122.060632)
Supplement: Supplementary file 1 [file cir-147-364-s001.pdf]

SUPPLEMENTAL MATERIAL

Myocardial involvement after hospitalization for COVID-19 complicated by troponin elevation: a prospective, multicenter, observational study

Jessica Artico[1*]MD, Hunain Shiwani[1*] BMBS, James C. Moon[1*]MD, Miroslawa Gorecka[2]MB, Gerry P. McCann[3]MD, Giles Roditi[4]MD, Andrew Morrow[4]PhD, Kenneth Mangion[4] PhD, Elena Lukaschuk[5] MSc, Mayooran Shanmuganathan[5] MBBS, Christopher A. Miller[6]PhD, Amedeo Chiribiri[7]PhD, Sanjay K. Prasad[8]MD, Robert D. Adam[1]MBBS, Trisha Singh[9]MBBS, Chiara Bucciarelli-Ducci[7,10]PhD, Dana Dawson[11]PhD, Daniel Knight[12]MD, Marianna Fontana[12]PhD, Charlotte Manisty[1]PhD, Thomas A. Treibel[1]PhD, Eylem Levelt[2]PhD, Ranjit Arnold[3] MD, Peter W. Macfarlane[13]DSc, Robin Young[14]PhD, Alex McConnachie[14]PhD, Stefan Neubauer[5]MD, Stefan K. Piechnik[5]PhD, Rhodri H. Davies[1]PhD, Vanessa M. Ferreira[5]PhD, Marc R. Dweck[9]PhD, Colin Berry[4]PhD, Oxford Acute Myocardial Infarction OxAMI Study Investigators, COVID-HEART investigators[§], John P. Greenwood[2]PhD.

Affiliations

1. Institute of Cardiovascular Science, University College London, London, UK.
2. Institute of Cardiovascular and Metabolic Medicine, University of Leeds, Leeds, UK.
3. University of Leicester and The NIHR Leicester Biomedical Research Centre, Glenfield Hospital, Leicester, UK.
4. Institute of Cardiovascular and Medical Sciences and British Heart Foundation Glasgow Cardiovascular Research Centre, University of Glasgow, Glasgow, UK.
5. Division of Cardiovascular Medicine, Radcliffe Department of Medicine, Oxford Centre for Clinical Magnetic Resonance Research, British Heart Foundation Centre of Research Excellence, Oxford NIHR Biomedical Research Centre, University of Oxford, Oxford, UK.
6. Division of Cardiovascular Sciences, School of Medical Sciences, Faculty of Biology, Medicine and Health, University of Manchester, Manchester, UK.
7. School of Biomedical Engineering and Imaging Sciences, King's College London, BHF Centre of Excellence and the NIHR Biomedical Research Centre at Guy's and St. Thomas' NHS Foundation Trust, The Rayne Institute, St. Thomas' Hospital, London, UK.
8. National Heart and Lung Institute, Imperial College, London, UK.
9. University of Edinburgh and British Heart Foundation Centre for Cardiovascular Science, Edinburgh, UK.
10. Royal Brompton and Harefield Hospitals, London UK, Guys' and St Thomas NHS Trust, London, UK; Bristol Heart Institute, University Hospitals Bristol and Weston NHS Trust, Bristol, UK
11. Department of Cardiology, Aberdeen Cardiovascular and Diabetes Centre, Aberdeen Royal Infirmary and University of Aberdeen, Aberdeen, UK.
12. Division of Medicine, Royal Free Hospital, University College London, London, UK.
13. Electrocardiology Core Laboratory, Institute of Health and Wellbeing, University of Glasgow, Glasgow, UK
14. Robertson Centre for Biostatistics, Institute of Health and Wellbeing, University of Glasgow, Glasgow, UK.

* Equal first authors.

[§] COVID-HEART investigators listed in the supplement

Table of contents

List of investigators page 3

Supplementary methods page 4

- **Inclusion and exclusion criteria** page 4
- **Recruitment and data collection of Covid-19 patients at acute phase** page 4
- **Recruitment of controls** page 4
- **Consent procedures** page 4

Role of the funding source page 4

CMR protocol page 5

CMR analysis page 5

- **Volumes and function** page 5
- **LGE** page 5
- **Image analysis of T1- and T2-maps** page 6
- **Extracardiac CMR analysis** page 6

Supplementary figures page 8

Supplementary tables page 14

List of investigators

Chief Investigator: JP Greenwood; *Grant applicants*: JP Greenwood, GP McCann, C Berry, M Dweck, JC Moon, CM Miller, A Chiribiri, S Prasad, VM Ferreira, C Bucciarelli-Ducci, D Dawson; *Trial Analysis Group*: James C. Moon (chair), John P. Greenwood, Jessica Artico, Hunain Shiwani, Rhodri Davies, Vanessa M. Ferreira, Marc Dweck, Colin Berry, Giles Roditi, , Robin Young, Alex McConnachie, Bernard Kelly, Peter W. Macfarlane, Gerry P. McCann, Christopher A. Miller; *Trial sites*: Leeds Teaching Hospitals NHS Trust: John P. Greenwood, Eylem Levelt, Miroslawa Goreka, Kathryn Somers, Roo J. Byrom-Goulthorp, Michelle Anderson, Laura Britton, Fiona Richards, Laura M. Jones; University Hospitals of Leicester NHS Trust: Gerry P. McCann, Ranjit Arnold, Alastair Moss, Jude Fisher, Joanne Wormleighton, Kelly Parke, Rachel Wright, Jian Yeo; NHS Grampian: Dana Dawson, Judith Falconer, Valerie Harries, Paula Henderson; NHS Lothian: Marc Dweck, Trisha Singh, David Newby; Oxford University Hospitals NHS Foundation Trust: Vanessa M. Ferreira, Stefan Piechnik, Iulia Popescu, Elena Lukaschuk, Qiang Zhang, Mayooran Shanmuganathan, Stefan Neubauer, Betty Raman, Keith Channon, Catherine Krasopoulos, Claudia Nunes, Liliana Da Silva Rodrigues, Harriet Nixon, Athanasia Panopoulou, Alison Fletcher, Peter Manley; NHS Greater Glasgow and Clyde: Colin Berry, Kenneth Mangion, Andrew Morrow, Robert Sykes, Kirsty Fallon, Ammani Brown, Laura Kelly, Christopher McGinley, Michael Briscoe, Rosemary Woodward, Tracey Hopkins, Evonne McLennan, Nicola Tynan, Laura Dymock; Mid Yorkshire Hospitals NHS Trust: Peter Swoboda, Judith Wright, Donna Exley; Birmingham NHS Foundation Trust: Richard Steeds, Kady Hutton, Sonia MacDonald; University College London Hospitals NHS Foundation Trust: James C. Moon, Thomas Treibel, Jessica Artico, Abhishek Shetye; Manchester University NHS Foundation Trust: Christopher M. Miller, Christopher Orsborne, William Woodville-Jones, Susan Ferguson, Konstantinos Bratis; Liverpool Heart and Chest Hospital NHS Foundation Trust: Timothy Fairbairn, Michail Sionas, Peris Widdows, Pei Gee Chew, Christian Marsden, Tom Collins, Linsha George, Lisa Kearney; University Hospital Southampton NHS Foundation Trust: Andrew Flett, Simon Smith, Alice Zhenge, Jake Harvey, Liliana Inacio, Tomas Hanam-Penfold, Lucy Gruner; Royal Free London NHS Foundation Trust: Marianna Fontana, Yousuf S.K. Razvi, Jacolene Crause, Nina M. Davies, Jessica Artico, James T. Brown, Liza Chaco, Rishi Patel, Tushar Kotecha, Dan S. Knight; Northumbria Healthcare NHS Foundation Trust: Thomas Green, David Ripley, Maria Thompson; Guy's and St Thomas NHS Foundation Trust: Amedeo Chiribiri, Ugochi Akerele, Elna Cifra, Ebraham Alskaf, Richard Crawley, Adriana Villa; University Hospitals Bristol NHS Foundation Trust: Chiara Bucciarelli-Ducci, Angus K. Nightingale, Kim Wright, Esther D. Bonnick, Emma Hopkins, Jessy George, Linta Joseph; Imperial College Healthcare NHS Trust: Graham Cole, Kavitha Vimalesvaran, Nadine Ali, Caitlin R. Carr, Alexandra A.R. Ross, Clara King; Royal Brompton and Harefield NHS Foundation Trust: Sanjay Prasad, Zohreh Farzad, Sara A. Salmi, Kevin Kirby; Newcastle Upon Tyne Hospitals NHS Foundation Trust: Adam McDiarmid, Hannah J. Stevenson, Pamela S. Matsvimbo, Lency Joji, Margaret Fearby, Benjamin Brown; St George's University Hospitals NHS Foundation Trust: Nicholas Bunce, Robert Jennings, Vennessa Sookhoo, Shatabdi Joshi; Liverpool University Hospitals NHS Foundation Trust: Prathap Kanagala, Sandra Fullalove, Catherine Toohey, Kate Fenlon; The Royal Devon and Exeter Hospital Foundation Trust: Nicholas Bellenger, Jingzhou He, Sarah Statton, Nicola Pamphilon, Anna Steele, Claire Ball, Ann McGahey, Silvia Balma, Lynsey Wilkes, Katy Lewis, Michelle Walter; Swansea Bay University Health Board: Adrian Ionescu, Tishi Ninan, Suzanne Richards, Marie Williams; Lewisham and Greenwich NHS Trust: Khaled Alfakih, Samia Pilgrim; Barts Health NHS Trust: James C. Moon, Jessica Artico, George Joy, Charlotte H. Manisty, Ifza Hussain, Thomas Treibel.

Supplementary Methods

Inclusion and exclusion criteria

Inclusion criteria: hospitalised-recovering patient population (age ≥ 18 years), or those recently discharged from hospital, with a diagnosis of Covid-19 based upon either a pathology or radiology diagnosis, with cardiac biomarkers (troponin I or T) increased above the sex-specific upper reference limit of the local laboratory range.
Exclusion criteria: being unable or unwilling to consent, contraindication to CMR, pregnancy or breast-feeding.

Recruitment and data collection of Covid-19 patients at acute phase

Screening was performed at an individual hospital level, with participating hospitals cross-referencing all admissions with a positive Covid-19 status (pathology/radiology diagnosis), with serum troponin results.
The source data included hospital records, National Health Service (NHS) health and social care records, clinical and office charts, laboratory and pharmacy records as well as digital images from radiology (chest X-ray / computed tomography / CMR) and cardiology (echocardiography / angiography / CMR).

Clinical working diagnoses for raised troponin were extracted from the medical records by the research teams, during patient index admission, without knowledge of MRI results (which were performed prior to or shortly after hospital discharge). Teams were provided with a standard definition of Type 1 and Type 2 infarction1: a) Type 1 MI was defined as: the detection of a rise and/or fall of cTn with at least one value above the 99th percentile URL and with at least one of the following: - symptoms of acute myocardial injury - new ischemic electrocardiography changes - development of pathological Q waves - imaging evidence of new loss of viable myocardium or new regional wall motion abnormality in a pattern consistent with an ischemic aetiology - identification of a coronary thrombus by angiography including intracoronary imaging or by autopsy. b) Type 2 MI was defined as an MI occurring secondarily to myocardial ischaemia and an illness or process other than acute atherothrombosis. Possible mechanisms underlying this imbalance between myocardial oxygen demand and supply include coronary spasm, coronary embolism, coronary artery dissection, sustained tachyarrhythmia, severe bradyarrhythmia, severe hypertension, respiratory failure, shock, severe anaemia or hypotension.

Recruitment of controls

Contemporary patient cohorts acting as control data were acquired for comparative CMR analysis. These were derived from ethically approved local research studies from the recruiting centres for COVID HEART using the same CMR scanners (vendor and field strength). Control populations included:
1) A population matched for age and CVD risk factors, who have been hospitalised and are Covid (+) and Troponin(−) [e.g. Capturing MultiORgan Effects of Covid-19 (C-MORE) cohort, Oxford, UK; NCT04510025].
2) A population matched for age and CVD risk factors, who have tested Covid(−) and Troponin(−).
The control populations were recruited prospectively at five COVID HEART centres as part of on-going funded research (e.g. CISCO-19, PREDICT, C-MORE, OxAMI). As this is a longitudinal, observational study, co-enrolment with other UK Covid-19 studies/trials was permitted.

Consent procedures

All recruited patients gave written informed consent as per the international ethical and scientific quality standard of Good Clinical Practice (GCP). Patient information sheets for COVID-HEART were provided in English, and in up to 10 other languages commonly spoken in the UK (including: French, Portuguese, Polish, Urdu, Bengali, Punjabi, Gujarati, Hindi, Somali and Arabic), to facilitate engagement across a range of community groups.
When eligibility criteria were confirmed, medical staff or appropriately trained support staff acquired consent from patients after allowing as much time as necessary to consider the study, or at least 24 h, whilst the patient was either an in- or out-patient. All participants had the right to withdraw from the study at any point. The reasons for withdrawals were recorded. If consent was withdrawn, or if the patient were to become incapacitated, any data collected up to that point remained on file and was included in the analysis.

ROLE OF THE FUNDING SOURCE

The funder had no involvement in: study design; collection, analysis, and interpretation of data; writing of the report; or the decision to submit for publication. A writing/analysis group (CB,MD,RD,VF,JG,AM,GM,JM,JA,HS,SP,GR,RY) oversaw image analysis at 3 corelabs (Oxford, Barts, Glasgow) and data transfer to the independent Glasgow Clinical Trials Unit. All authors have read and accepted the final version of the manuscript.

CMR PROTOCOL

The main CMR protocol was in keeping with Society for Cardiovascular Magnetic Resonance (SCMR) recommended CMR protocols for scanning patients with active or convalescent phase Covid-19 , and took approximately 50 min to acquire, with an optional shortened and extended protocol dependent of patient preference and ability. An estimated glomerular filtration rate (eGFR) and haematocrit were measured prior to each CMR scan. For patients with significant renal failure (eGFR < 30 ml/min/1.73 m2), late gadolinium enhancement (LGE) and post-contrast T1-mapping were omitted and a contrast-free CMR scan performed. The main CMR study protocol included the following components and typical parameters, which may vary by vendor and field strength, but remain comparable overall.

A. Localiser sequences and breath-hold transverse Half-Fourier Acquisition Single-shot Turbo spin Echo (HASTE) imaging stacks covering lung and abdomen to 1–2 cm below the kidneys. Typical sequence parameters: TE 1.33ms, TR 700ms, slice thickness 8 mm, FOV = 400 mm, FOV phase 100%, flip angle 10°.

B. Cine images acquired with breath-hold balanced steady-state free precession (bSSFP) sequence. Long axis views of the LV: 4-chamber, 2-chamber, and 3-chamber views. Ventricular short-axis stack. Sequence parameters matching the cine image acquisition in long-axis. Sequence parameters: TE 1.05ms, TR 40.29ms, slice thickness 8 mm, 25% distance factor, FOV = 500 mm, FOV phase 75%, flip angle 50°.

C. Native (pre-contrast) Myocardial T1 and T2 mapping were acquired in 3 short-axis cuts of the LV (basal, mid-ventricular, apical) using a single breath-hold shortened modified Look-Locker inversion (ShMOLLI) 5(1)1(1)1 technique, , where available. Shimming was performed to avoid artefacts. Native T1-mapping was acquired in 3 short-axis cuts of the LV (basal, mid-ventricular, apical) to match the locations of segments 1–16 of the American Heart Association 17-segment model. he apical segment 17 was omitted. Typical pulse sequence parameters: TE 1.07ms, TR 379ms, slice thickness 8 mm, FOV = 360 mm FOV phase 75%, flip angle 35°, distance factor 25%, generalised auto-calibrating partially parallel acquisition (GRAPPA) 2 with 24 reference lines.

D. Native (pre-contrast) T2-mapping: matching in slice location to the T1 maps, was acquired using either a T2-prepped b-SSFP sequence with a minimum of 3 source images (e.g. MyoMaps T2-mapping for Siemens scanners), or a black-blood prepared, navigator-gated, free-breathing hybrid gradient (echo planar imaging, EPI) and spin-echo multi-echo sequence (GRASE). Typical sequence parameters: TE 1.3ms, TR 222.43ms, slice thickness 8 mm, FOV = 360 mm, FOV phase 80%, flip angle 20°.

E. LGE images were acquired ~ 5–15 min after intravenous injection of 0.1–0.15 mmol/kg of gadolinium-based contrast agent (GBCA, accepted GBCA agents include: gadobutrol and gadoteric acid.), with a free-breathing phase-sensitive motion correction bSSFP or breath-hold, segmented inversion-recovery sequence. Contiguous stack of LV short-axis images and single long-axis slices in 2-chamber, 4-chamber and 3-chamber at the same slice locations as obtained for cine imaging were acquired. A Look-Locker sequence was used to determine the appropriate inversion time (TI) .

F. Post-contrast T1 measurements were acquired at the exact same locations as the native T1-maps and performed at least 10 min after injection of GBCA, using the same pulse sequence and parameters as the native T1-maps.

CMR ANALYSIS

CMR data sets were analysed within a disseminated core-lab (Barts structure/function/LGE; Oxford mapping; Glasgow extracardiac anatomy). Results were discussed on a weekly basis by a disseminated panel of experts.

Volumes and function

Left ventricular (LV) structure and function was analysed using a clinically validated artificial intelligence (AI) platform. Right ventricles and atria were analysed manually, in detail, trabeculations of the RV were ignored, and a smooth endocardial border was drawn to improve reader reproducibility.

LGE

A standard operating procedure was developed for late gadolinium enhancement (LGE), with all analyses performed by three experienced independent observers using Circle CVI42 version 5.13.5 (Circle Cardiovascular Imaging Inc., Calgary, Alberta, Canada). Furthermore, all LGE analysis were reviewed by two level 3 accredited CMR supervisors. LGE was defined as area of hyperintense signal in the myocardium on the PSIR images. The presence of LGE in most cases was obvious but where there was uncertainty about the presence of LGE, all long axis LGE, contiguous SAX slices, cine images or, where available, dark blood LGE were reviewed to confirm the results. LGE was qualitatively evaluated as : A)infarct, subendocardial or transmural scar conforming to a coronary territory; B)non-ischaemic, subepicardial or intramyocardial scar- a category that includes most myocarditis related scar; C)Both A and B, dual pathology; D)micro-infarction, bright subsegmental areas of scar sometimes in multiple territories; E)likely pre-existing scar (where there is a known disease causing a certain pattern of LGE e.g. amyloid, sometimes with prior imaging) or nonspecific scar; F)no scar (figure 2 and Figure S1).

Furthermore, in scans considered to have LGE, a quantitative LGE analysis was performed by semi-automated signal intensity analysis. Epicardial then endocardial contours were manually drawn, with care taken to exclude artefacts, blood pool, fat and pericardium. An area of normal remote myocardium (the darkest area of myocardium throughout the whole SAX stack) was defined alongside identification of an area with visually the greatest increased signal

intensity. LGE was then obtained using the 5SD techniques are recorded. The percentage of LGE by these techniques was then multiplied by the absolute LV mass on cine images to determine LGE mass.
Quantitative assessment was performed by semi-automated signal intensity analysis using a 5-SD approach [1] 25. LGE of <1% of the myocardium at the right ventricular (RV) insertion points only was ignored.

Image analysis of T1- and T2-maps
Global T1, T2 and ECV analyses were performed blinded to the clinical information on commercially-available post-processing software (cmr42, Circle Cardiovascular Imaging, Calgary, Alberta, Canada, v5.10). Endo- and epicardial contours were manually placed within the left ventricular myocardium on short-axis T1, T2-maps, with care to avoid partial volume with surrounding tissue, such as fat or blood pool. Raw images were examined for breathing or cardiac motion, or SSFP artefacts. Where available, quality control maps for 'goodness of fit' were assessed for image quality as previously published. Individual average T1, T2 and ECV results were marked with a quality score depending on the data quality: 0 – good quality; 1 - minor issues, <50% myocardial segments affected; 2 - major issues, >50% image data corrupted; 3 – not analysable or missing data. Scores 0-1 were considered reliable and adequate reportable myocardial coverage. Conversely, the estimates with the quality scores 2-3 were recommended for rejection from the final statistical analysis. For ECV quantification, additional regions of interest were also placed in LV blood pool in both the native and post-Gd T1-maps, avoiding artefacts and papillary muscles. ECV was calculated from the native and post-contrast T1 in accordance with the previously published formula:

$$ECV = (1 - \text{haematocrit}) \times (\Delta R1\text{myocardium}/\Delta R1\text{blood}), \text{ where } R1 = 1/T1.$$

Harmonisation: Mapping image analysis for global T1/T2/ECV values was performed by a single image analyst (EL) with over 5 years' experience in mapping image analysis and over 10 years in CMR data analysis. The observer has undergone the standardised training; complex datasets were referred for internal reviews within the OCMR core lab (SKP, VMF). For the different sites, scanners and/or sequences, local normal ranges for T1- and T2-mapping were obtained directly from the sites according to SCMR guidelines, or ShMOLLI T1-mapping norms were used after the sequence conformance was validated using phantoms, as appropriate. The normal ranges were all expressed as normal mean ± SD for each site. In line with prior observation, the variation in the reported normal SDs between sites was not suitable for the application of z-scores; instead, the normalised nT1 and nT2 were reported, obtained by dividing the individual T1 and T2 measurements by the corresponding normal mean value for the appropriate site/scanner/sequence combination. In line with prior studies, ECV was deemed to have a satisfactory intrinsic compensation for inter-scanner variability, and thus was used directly.

Where T2 mapping was available matching a slice with LGE, a ROI was drawn to sample the T2 values in both the area of LGE and in remote myocardium and the difference calculated as ΔT2 (T2 in the LGE area – T2 in the remote myocardium).

EXTRA-CARDIAC CMR ANALYSIS
The extracardiac CMR analysis were performed by an experienced Consultant Radiologist with 20+ years' experience in cardiovascular & thoracic radiology. Findings were divided into those potentially related to Covid -19 itself (i.e. the manifestations of pneumonitis or heart failure) and those that are truly incidental but potentially important for patient care. In particular, they were divided into the following sections:

1 - Potential Covid-19 Related –
1A Pneumonitis Related Changes - graded as
0 - No abnormality
1 - Minor peripheral pulmonary signal changes, predominantly posterior and thought potentially due to either Covid-19 pneumonitis or just dependent change
2 - Peripheral pulmonary signal changes being greater than grade 1 and peripheral, patchy, non-dependent thought likely to be due to Covid-19 pneumonitis in pandemic scenario
3 - Extensive pulmonary signal changes compatible with severe Covid-19 pneumonitis in pandemic scenario.

Modifiers - narrative for findings such as lobar collapse, cavitation etc. which were unusual

1B Additional Pulmonary Findings potentially Covid-19 related -

Pleural Effusion - graded as 0 = Absent; 1 = Small effusion(s); 2 = Moderate effusion(s); 3 -= Large effusion(s)
Pulmonary Embolism/Thrombosis - graded as - 0 = absent, 1 = present and if so then add narrative description
Pulmonary Venous Thrombosis - graded as - 0 = absent, 1 = present and if so then add narrative description
Pericardium - graded as - 0 = Normal, 1 = Abnormal thickening/enhancement
Pericardial Effusion - graded as - Absent, Small (< 10mm depth), Medium, Large (>20 mm depth) (0,1, 2, 3)

2 - Potentially Clinically Significant Pre-Existing Cardiac Incidental Findings -

Single free text field for a narrative description of anything potentially important spotted e.g. valvular stenosis &/or incompetence, intracardiac thrombus, congenital abnormality, obvious chamber abnormality, features of cardiomyopathy such as focal hypertrophy, sternal wires, valve replacements etc.

3 - Potentially Clinically Significant Incidental Extracardiac Findings -

Single free text field for a narrative description of anything potentially important spotted, the following were thought the most likely scenarios (although in real terms unlikely) (-

Lung or pleural mass - suspicion of cancer; Aortic Aneurysm; Mediastinal mass - e.g. lymphadenopathy; Skeletal abnormality - suspicion of cancer; Upper abdominal organ - suspicion of cancer or potentially important benign condition (e.g. hepatic or renal masses, gallstones and similar); Abnormally raised diaphragm

N.B. Small hiatus hernias, small hepatic and renal cysts, colonic diverticulosis and similar common findings of little clinical importance were not recorded (only large hiatus hernias and abnormal/complex looking cysts).

Supplementary results:

Extracardiac findings

The Covid+/Troponin+ patients had residual lung abnormalities (33%), pleural effusions (13%) and pericardial effusions (14%), at higher frequency than other groups, table S6.

Figure S1: Patterns of LGE in Covid+/Troponin+ patients

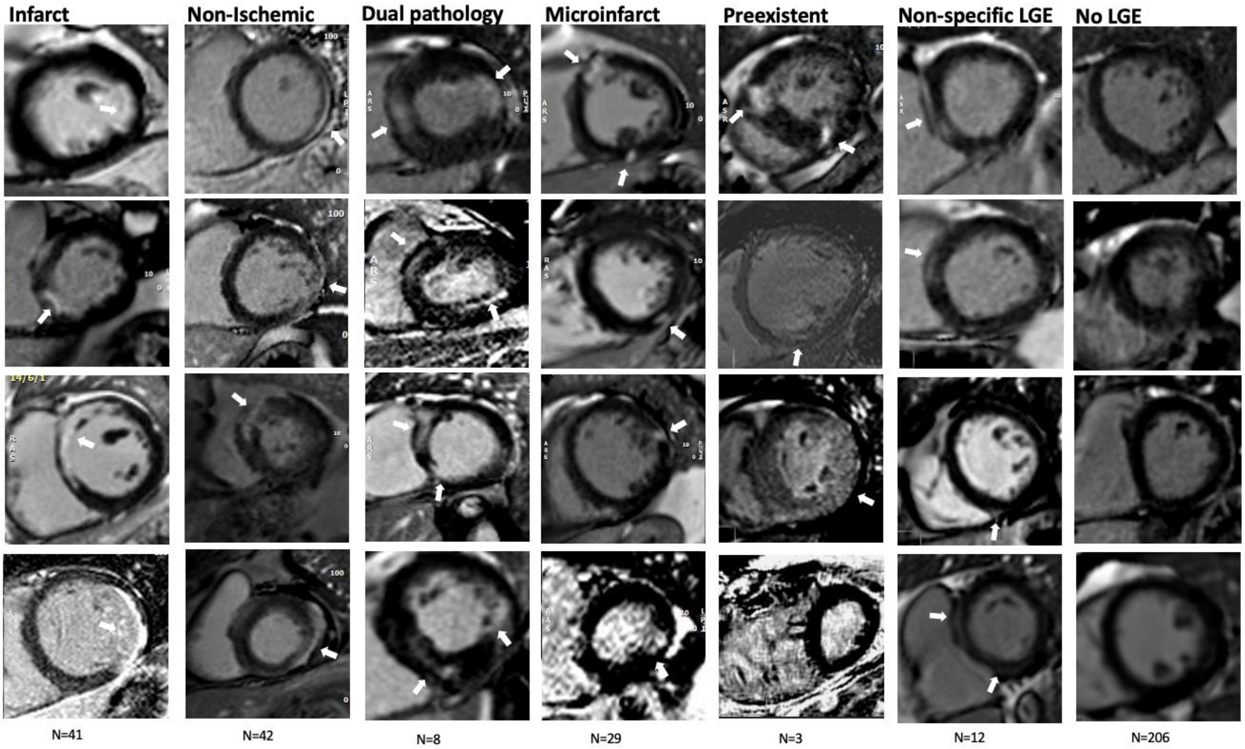

Patterns of LGE: A) Infarct (bright, subendocardial, territorial); B) Non-ischemic (mid myocardial, less bright, more diffuse); C) Dual pathology (both A and B); D) Microinfarcts (bright spots of subsegmental LGE, up to 10 grams, often but not exclusively subendocardial and potentially in more than one territory); E1) Chronic, known preexistent disease (in just 4 cases: hypertrophic cardiomyopathy, dilated cardiomyopathy, cardiac amyloidosis, pulmonary hypertension) E3) Non-specific (unequivocal LGE that both cannot be considered normal but insufficient to assign with certainty to any other category). F) No or trivial LGE (minor RV insertion point, trabecular or septal perforator LGE being therefore ignored).

LGE: Late Gadolinium Enhancement

Figure S2: Patterns of LGE in controls

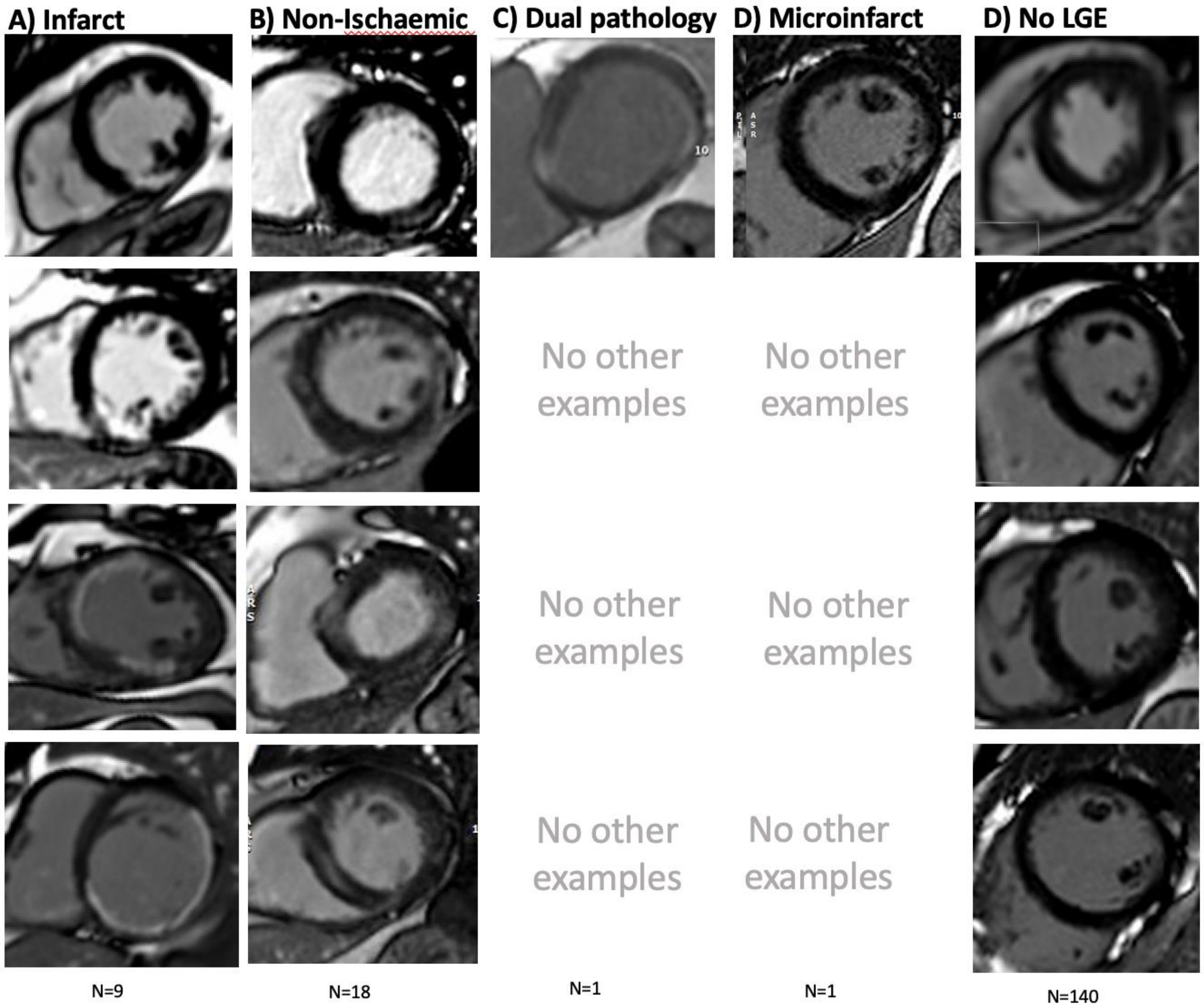

Patterns of LGE in controls (in brackets the features of each): A) Infarct (bright, sub-endocardial, territorial); B) Non-ischaemic (mid myocardial, less bright, more diffuse); C) Dual pathology(both a and b); D) Microinfarcts (bright spots under half a segment, often a few grams of LGE often but not exclusively subendocardial and potentially in more than one territory); E) No LGE or non-significant LGE (minor RV insertion point LGE alone or trabecular LGE alone or septal perforator LGE alone, that can be considered normal).

LGE: Late Gadolinium Enhancement

Figure S3: Example late gadolinium enhancement quantification

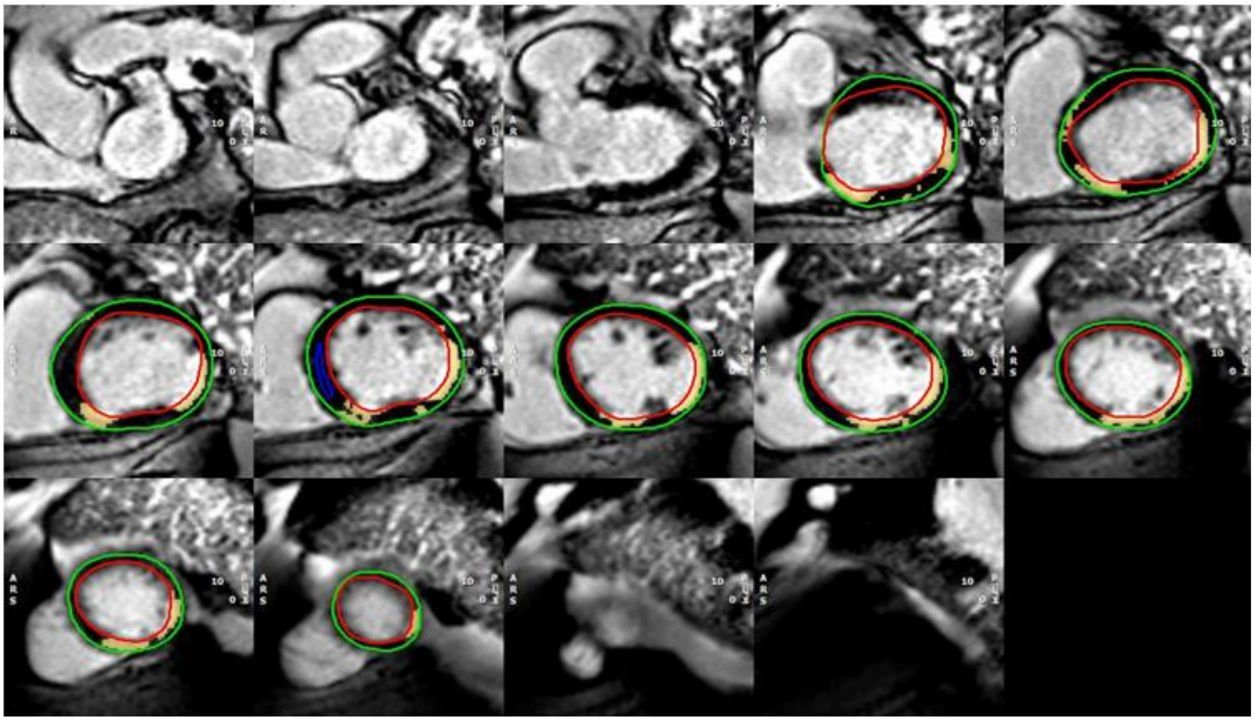

A single case, one of the 519 in this study with corelab LGE quantification. Here with red: endocardium; green: epicardium. The blue contour on slice 7 is defining the standard deviation of remote myocardium permitting the yellow pixels to be defined (signal >5 standard deviations above remote).

Figure S4 Normalized T1, Normalized T2 and Global Extracellular volume values in the three populations.

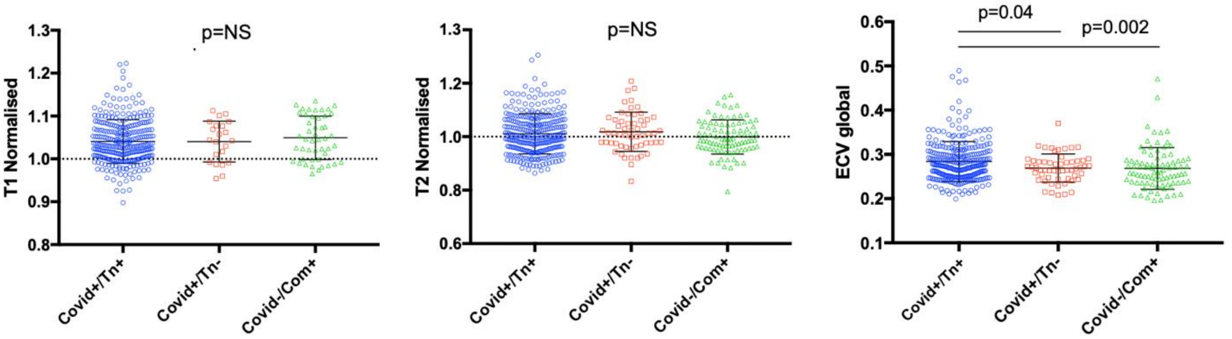

ECV=Extracellular volume

Figure S5. Percentage of patients with LGE at cardiac MRI divided by extent of Left ventricular Systolic Dysfunction.

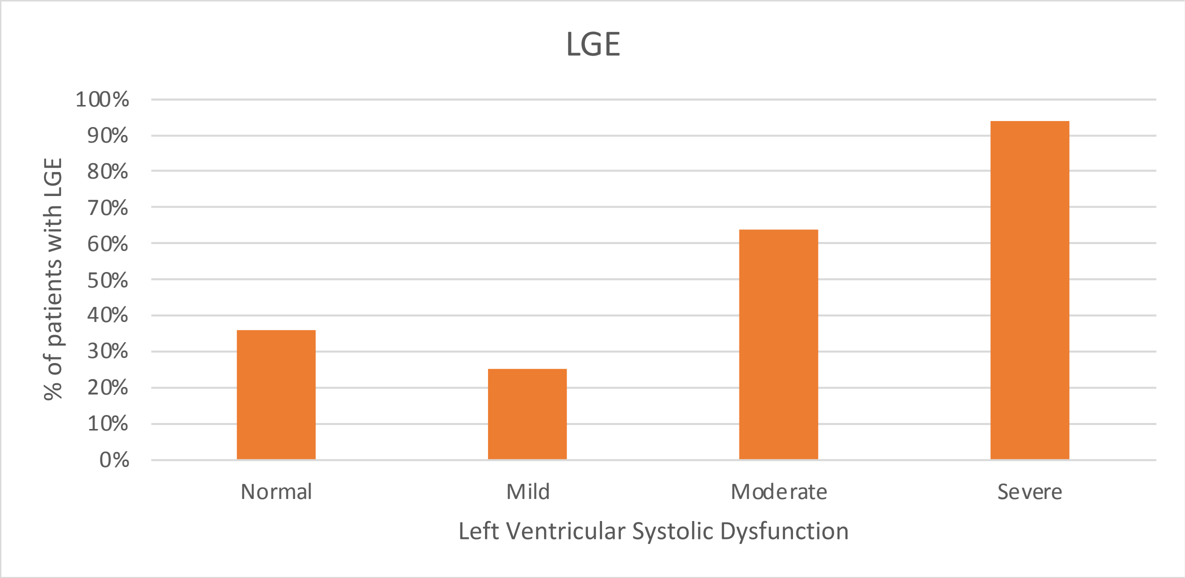

Normal (above age/sex reference range), mild (below age/sex reference range to 50%), moderate (50-40%), severe (<40%)

LGE: Late Gadolinium Enhancement

Figure S6

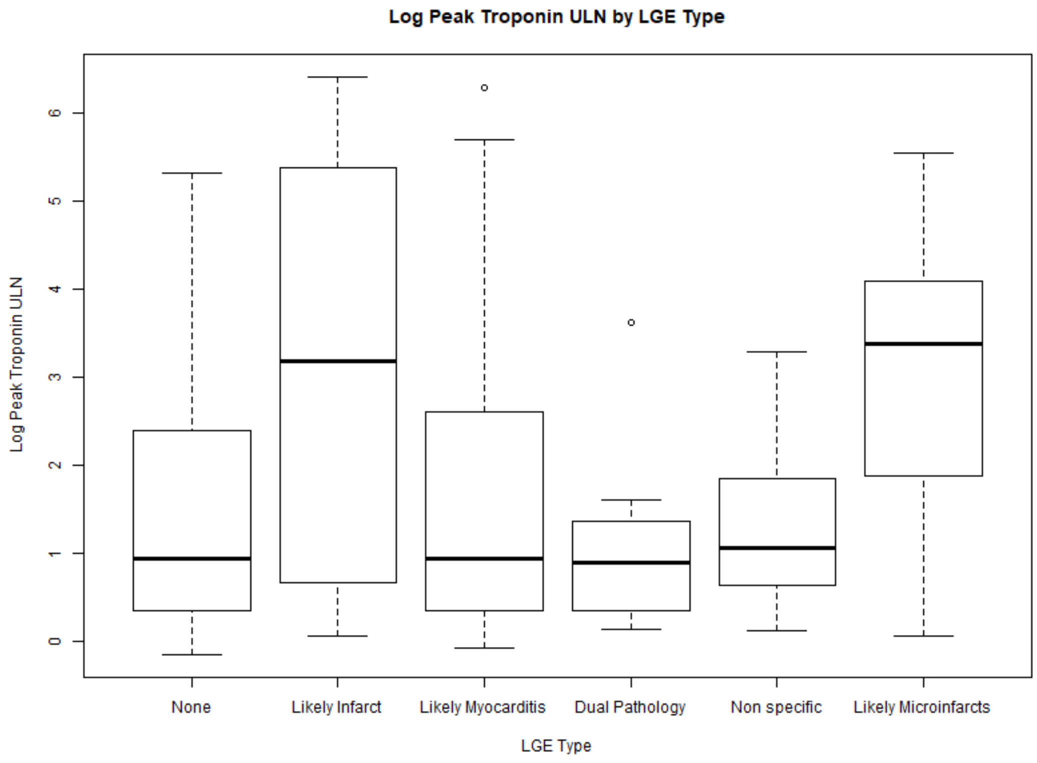

Figure S7: Patient level excess cardiac pathology

	Covid + / Troponin +	Covid + / Troponin -	Covid - / Comorbidities
Cardiac abnormalities	61%	36%	31%
LV Impairment	17%	3%	7%
RV Impairment	59%	57%	37%
Any Scar	42%	7%	23%
Scar Pattern			
Infarct	13%	2%	7%
Micro-infarct	9%	0%	1%
Non-Ischaemic	13%	5%	14%
Lake-Louise probable recent myocarditis	7%		2%

1. *Covid+/Troponin+ have high rates of comorbidities*
2. *Comorbid control scar - mainly non-ischaemic*
3. *Covid+/Troponin+ scar - mainly infarct/microinfarct*
4. *Much myocarditis scar likely pre-existant*

COVID related cardiac injury:

More thrombosis related (macro/microangiopathic)

Less myocarditis related

Legend: LV=left ventricle; RV= right ventricle

Table S1: Clinical characteristics

	Total population Covid+/ Troponin+ (n=383)	MRI population Covid+/ Troponin+ (n=342)
Age (years), (median IQR)	62.2 [54.0, 70.6]	61.3 [53.4, 69.7]
Male Sex (n, %)	263(68.7%)	243(71.1%)
BMI (kg/m^2) (median IQR)	29.1 [25.4, 33.2]	29.0 [25.1, 33.2]
BSA (m^2) (median IQR)	2.0 [1.8, 2.1]	2.0 [1.8, 2.1]
Ethnicity (n, %)		
Indian	13(3.4%)	12(3.5%)
Pakistani	9(2.4%)	6(1.8%)
Bangladeshi	5(1.3%)	4(1.2%)
Black Caribbean	12(3.1%)	11(3.2%)
Black African	23(6.0%)	22(6.4%)
Chinese	4(1.0%)	4(1.2%)
Other Asian	17(4.5%)	16(4.7%)
Other ethnic group/mixed ethnicity	42(11.0%)	41(11.0%)
Timing		
Length of hospital stay (days, median(IQR))	9.0(5.0,18.0)	9.0(5.0,16.0)
Diagnosis to MRI (days, (median IQR))	-	32.0 [25.0, 42.0]
Admission to MRI (days, median(IQR))	-	30.0 [21.0, 38.0]
Discharge to MRI (days, median(IQR))	-	21.0 [11.0, 27.0]
**Covid diagnosis and treatment**		
Covid diagnosis by positive PCR test, n(%)	364(95.5%)	325(95.0%)
Covid diagnosis by positive Antibody test, n(%)	18(4.7%)	17(5.0%)
Covid diagnosis on CXR/CT, n(%)	273(71.7%)	246(71.9%)
Community acquired, n(%)	313(82.2%)	276(80.7%)
Hospital acquired, n(%)	24(6.3%)	24(7.0%)
Oxygen therapy, n(%)	300(78.7%)	265(77.5%)
Non-invasive respiratory ventilation, n(%)	97(25.5%)	88(25.7%)
Invasive ventilation, n(%)	38(10.0%)	34(9.9%)
ECMO, n(%)	1(0.3%)	1(0.3%)
Inotropes, n(%)	31(8.1%)	29(8.5%)
Dialysis, n(%)	7(1.8%)	7(2.0%)
Steroid therapy, n(%)	218(57.2%)	190(55.6%)
Anti-viral therapy, n(%)	71(18.6%)	62(18.1%)
Hydroxychloroquine, n(%)	1(0.3%)	1(0.3%)
**Past medical history**		
Prior MI/ACS, n(%)	39(10.2%)	34(9.9%)
Revasc (PCI/CABG), n(%)	36(9.4%)	31(9.1%)
Hypertension, n(%)	175(45.9%)	160(46.8%)
Valvular HD, n(%)	18(4.7%)	15(4.4%)

Heart failure, n(%)	30(7.9%)	22(6.4%)
Arrhythmia, n(%)	43(11.3%)	29(8.5%)
Congenital Heart Disease, n(%)	2(0.5%)	2(0.6%)
Peripheral Vascular Disease, n(%)	12(3.1%)	8(2.3%)
Diabetes (type 1), n(%)	6(1.6%)	6(1.8%)
Diabetes (type 2), n(%)	93(24.4%)	78(22.8%)
CVA, n(%)	17(4.5%)	15(4.4%)
COPD, n(%)	38(10.0%)	28(8.2%)
Moderate-severe CKD, n(%)	20(5.2%)	16(4.7%)
Cancer, n(%)	20(5.2%)	18(5.3%)
Smoking history, n(%)		
Never	231(60.6%)	215(62.9%)
Former	127(33.3%)	106(31.0%)
Current	23(6.0%)	21(6.2%)
Charlson score		
Median (IQR)	3.0(1.0,4.0)	2.0(1.0,3.0)
Range	(0.0,10.0)	(0.0,10.0)
QRISK3 score		
Median (IQR)	15.9(8.9,26.4)	15.7(8.4,24.6)
Range	(0.0,81.4)	(0.0,68.8)
**Admission Medications**		
Anti-platelet (Aspirin/Clopidogrel/Ticagrelor), n(%)	81(21.3%)	75(21.9%)
Statin, n(%)	169(44.4%)	146(42.7%)
Beta-Blocker, n(%)	90(23.6%)	78(22.8%)
Anticoagulant (warfarin/DOAC), n(%)	32(8.4%)	22(6.4%)
ACEI/Angiotensin Receptor Blocker, n(%)	135(35.4%)	121(35.4%)
Mineralocorticoid (spironolactone/eplerenone, n(%)	11(2.9%)	11(3.2%)
Oral hypoglycaemic agents, n(%)		
SGLT2 inhibitor	11(2.9%)	9(2.6%)
GLP1 antagonist	4(1.0%)	2(0.6%)
Insulin, n(%)	24(6.3%)	22(6.4%)
**Discharge Medications**		
Anti-platelet (Aspirin/Clopidogrel/Ticagrelor), n(%)	128(33.8%)	120(35.1%)
Statin, n(%)	206(54.4%)	185(54.1%)
Beta-Blocker, n(%)	171(45.1%)	158(46.2%)
Anticoagulant (warfarin/DOAC), n(%)	134(35.4%)	113(33.0%)
ACEI/Angiotensin Receptor Blocker, n(%)	171(45.1%)	156(45.6%)
Mineralocorticoid (spironolactone or eplerenone), n(%)	28(7.4%)	25(7.3%)
Oral hypoglycaemic agent, n(%)		
SGLT2 inhibitor	8(2.1%)	6(1.8%)
GLP1 antagonist	2(0.5%)	0(0.0%)

Insulin, n(%)	48(12.7%)	42(12.3%)
NSAIDs, n(%)	10(2.6%)	9(2.6%)
Colchicine, n(%)	13(3.4%)	13(3.8%)
Steroids, n(%)	63(16.6%)	54(15.8%)
**Pathology results**		
Haemoglobin, g/L, Mean (SD)	136.5(20.4)	137.0(18.9)
Platelets, 10^3/µL Mean (SD)	250.7(108.8)	251.7(109.4)
Packed cell volume (haematocrit), %, Mean (SD)	0.4(0.1)	0.4(0.1)
White Cell Count, 10^3/µL Mean (SD)	10.2(11.8)	10.0(10.9)
Neutrophils, 10^3/µL, Mean (SD)	8.2(8.9)	8.1(8.4)
Creatinine, µmol/L Mean (SD)	102.6(67.3)	102.1(69.2)
Alanine Aminotransferase, U/L, Mean (SD)	55.3(91.2)	57.4(95.1)
Alkaline Phosphatase, U/L, Mean (SD)	90.4(56.0)	90.7(58.1)
Peak CRP, mg/L, Mean (SD)	130.8(114.5)	129.7(117.0)
Peak BNP, pg/mL, Mean (SD)	383.4(320.1)	383.4(320.1)
Peak Troponin I (xULN) (median IQR)	5.6(2.0,48.7)	8.3(2.0,51.2)
Peak Troponin T (xULN) (median IQR)	2.7(1.4,12.4)	2.8(1.4,12.4)
Peak D-dimer, ng/mL, Mean (SD)	4401.2(11809.3)	4642.6(12333.8)
INR, Mean (SD)	1.2(0.8)	1.2(0.8)
Prothrombin time, sec, Mean (SD)	13.4(13.4)	13.4(14.0)
Peak fibrinogen, g/L, Mean (SD)	10.3(43.0)	10.8(45.2)

Legend: ACEi: Angiotensin Converting Enzyme inhibitors; ACS: Acute Coronary Syndrome; BMI: Body Mass Index; BNP: Brain Natriuretic Peptide; BSA: Body Surface Area; CABG: Coronary Artery Bypass Graft; CKD: Chronic Kidney Disease; MRI: Cardiac Magnetic Resonance Imaging; COPD: Chronic Obstructive Pulmonary Disease; CRP: C-reactive Protein; CT: Computed Tomography; CVA: Cerebrovascular Accident; CXR: Chest X-Ray; GLP-1: Glucagon Like Peptide-1; INR: International Normalized Ratio; ECMO: Extracorporeal Membrane Oxygenation; NSAIDs: Non-steroidal Anti-inflammatory drugs; MI: Myocardial Infarction; PCI: Percutaneous Coronary Intervention; SGLT2: Sodium Glucose Cotransporter-2.

-

Table S2: Coronary angiography results (65 patients)

Variable		
Normal coronary arteries (no plaque)	N (%)	21 (33.9%)
Non-obstructive plaque	N (%)	21 (33.9%)
Obstructive plaque	N (%)	23 (37.1%)
Left main stem stenosis	N (%)	4 (6.5%)
Coronary intervention	N (%)	23 (37.1%)

Table S3: Working diagnosis for raised troponin

Clinical diagnosis according to Universal Definition of MI	Number	Cardiac MRI diagnosis						
		No scar	Likely Infarct	Likely Myocarditis	Dual Pathology	Non-specific scar	Likely Microinfarcts	No LGE available
Type 1 MI	60	13	23	2	0	1	17	4
Type 2 MI	10	9	0	0	1	0	0	0
Cardiac cause of non-ischaemic myocardial injury								
Heart failure / cardiomyopathy	6	3	0	1	0	0	1	1
Myocarditis	14	9	0	3	0	0	1	1
Non-cardiac cause of non-ischaemic myocardial injury*	81	55	4	12	1	5	1	3
Multiple aetiologies	57	35	3	10	1	2	5	1
No clinical diagnosis for troponin elevation	114	66	11	14	5	7	4	7

*includes systemic conditions such as sepsis, renal failure.

Table S4 MRI data according to LGE presence in each group, showing that LGE presence is associated with worse measured parameters in all groups

	Covid+/Troponin+ where LGE available			Covid+/Troponin- Where LGE available			Covid-/ Comorbidity+ Where LGE available		
	n=342			n=64			n=113		
	LGE -	LGE +	p-value	LGE -	LGE +	p-value	LGE -	LGE +	p-value
**N of patients**	190 (58%)	135 (42%)		55 (93%)	4 (7)		84 (77%)	25 (23%)	
**LVEDV (mL)**	145±36	175±69	**<0.001**	146±35	171±46	0.19	144±35	199±37	**<0.001**
**LVESV (mL)**	50±23	78±61	**<0.001**	52±19	65±38	0.23	49±18	89±39	**<0.001**
**LVM (g)**	111±34	126±42	**<0.001**	98±25	114±18	0.22	99±26	125±20	**<0.001**
**LVEF (%)**	66±9	58±14	**<0.001**	65±7	64±9	0.77	66±8	56±13	**<0.001**
**RVEDV (mL)**	156±44	157±46	**0.05**	159±36	190±61	0.17	156±45	188±43	**0.002**
**RVESV (mL)**	72±26	84±38	**<0.001**	74±22	96±37	0.11	69±28	90±22	**0.001**
**RVEF (%)**	55±8	51±11	**<0.001**	54±6	50±9	0.27	60±7	52±7	**0.005**
**LAESA 4C (cm2)**	26±6	28±9	**0.03**	23±6	26±5	0.35	**22±6**	27±6	<0.001
**LGE, %**	-	9±14	**-**	-	5±4	-	**-**	9±8	-
**LGE, grams**	-	11±11	**-**	-	6±6	-	**-**	11±10	-

Legend: LAESA 4C: Left Atrial End Systolic Area in 4 chamber; LVEDV: Left Ventricular End Diastolic Volume; LVEDVi: Left Ventricular End Systolic Volume LVEF: Left Ventricular Ejection Fraction; LVM: Left Ventricular Mass, RVEDV: Right Ventricular End Diastolic Volume; RVESV: Right Ventricular End Systolic Volume; RVEF: Right Ventricular Ejection Fraction.

Table S5: MRI data divided by disease severity

	No Support	Only Oxygen	Non-Invasive Ventilation	Invasive Ventilation	P-value
N of patients	76	169	63	34	
LVEDVi (mL/m2)	84±20	81±32	74±21	74±17	0.07
LVEF (%)	61±13	63±12	63±13	65±9	0.54
RVEF (%)	54±9	52±10	51±9	55±7	0.16
RVEDVi (mL/m2)	83±18	80±21	81±28	77±15	0.46
LGE n (%)	34 (47%)	79 (49%)	16 (28%) #	6 (19%) *#	**0.002**

Legend: LGE: Late Gadolinium Enhancement; LVEDVi: Left Ventricular End Diastolic Volume indexed; LVEF: Left Ventricular Ejection Fraction; RVEDVi: Right Ventricular End Diastolic Volume indexed; RVEF: Right Ventricular Ejection Fraction. *=p<0.05 vs no Support
#=p<0.05 vs only Oxygen

Table S6: Extra-cardiac findings

Extracardiac findings		Covid+/ Troponin+ n=342	Covid+/ Troponin- n=64	Covid-/Comorbidity+ n=113	p value vs Covid+/ Troponin-	p value vs Covid-/ Comorbidity+	global p-value
**Pneumonitis**	Nothing visible on MRI	189 (55%)	49 (86%)	108 (96%)	*	*	<0.001
	Dependent/Basal signal only	41 (12%)	5 (9%)	4 (4%)	NS	*	
	Non-Dependent/probable covid	80 (24%)	3 (5%)	0 (0%)	*	*	
	Extensive pulmonary signal change affecting all lung zones	31 (9%)	0 (0%)	0 (0%)	*	*	
**Pleural Effusion**	None	299 (87%)	57 (100%)	111 (99%)	*	*	0.04
	Small	29 (9%)	0 (0%)	1 (1%)	NS	*	
	Moderate	10 (3%)	0 (0%)	0 (0%)	NS	NS	
	Large	4 (1%)	0 (0%)	0 (0%)	NS	NS	
**Pericardial Effusion**	None	300 (88%)	56 (98%)	109 (97%)	NS	*	0.03
	Small	34 (10%)	1 (2%)	2 (2%)	NS	*	
	Moderate	7 (2%)	0 (0%)	1 (1%)	NS	NS	
	Large	1 (1%)	0 (0%)	0 (0%)	NS	NS	
**Abnormal Pericardium**		1 (1%)	0 (0%)	0 (0%)	NS	NS	NS
**Pulmonary Embolism**		1 (1%)	0 (0%)	0 (0%)	NS	NS	NS

*=p<0.005 for multiple comparison.
